# Nutritional Supplements for Muscle Hypertrophy: Mechanisms and Morphology—Focused Evidence

**DOI:** 10.3390/nu17223603

**Published:** 2025-11-18

**Authors:** Andreea Maria Mănescu, Simona Ștefania Hangu, Dan Cristian Mănescu

**Affiliations:** Faculty of AgriFood and Environmental Economics, Bucharest University of Economic Studies, 010374 Bucharest, Romania

**Keywords:** resistance training, skeletal muscle, muscle protein synthesis, leucine threshold, creatine monohydrate, HMB, omega-3 fatty acids, citrulline, collagen peptides, ultrasound, MRI

## Abstract

Nutritional supplementation is widely used in resistance training, yet assessment of “hypertrophy” is often confounded by body-composition surrogates. This narrative review, anchored in mechanistic plausibility, integrates trials reporting morphology-direct outcomes (ultrasound/MRI). Across 46 eligible trials, protein/essential amino acids (EAA) showed consistent benefits when daily intake was <1.6 g·kg^−1^·day^−1^ or when per-meal leucine provision was <2–3 g; effects plateaued once intakes exceeded ~2.0 g·kg^−1^·day^−1^. Creatine monohydrate (3–5 g·day^−1^, with or without loading) produced measurable increases in muscle thickness or cross-sectional area in interventions lasting ≥8–12 weeks, mediated by enhanced training volume and quality. β-hydroxy-β-methylbutyrate (HMB, 3 g·day^−1^) demonstrated conditional utility during high training stress or caloric deficit, but was largely neutral in well-fed, resistance-trained cohorts. Adjuncts such as omega-3 fatty acids (1–2 g·day^−1^), citrulline (6–8 g pre-exercise), and collagen (10–15 g·day^−1^ plus vitamin C) primarily facilitated training tolerance, recovery, or connective-tissue adaptation, rather than driving hypertrophy directly. A tiered model is proposed: protein/EAA as the foundation, creatine as amplifier, HMB as conditional agent, and adjuncts as facilitators. Methodological heterogeneity, short intervention length, and inconsistent imaging protocols remain limiting factors, underscoring the need for standardized ultrasound/MRI and adequately powered, preregistered trials.

## 1. Introduction

Nutritional supplementation is widely adopted in resistance training and bodybuilding communities [1,2,3,4], because even small improvements—some of which may be produced by supplementation—in training quality, recovery, or translational efficiency can lead to measurable increases in muscle size. This topic matters beyond sport performance because skeletal muscle contributes to metabolic health, functional capacity, and injury resilience across the lifespan [5,6,7,8]. Interpretation of this literature is challenging because study designs, participant training status, dosing strategies, and—critically—the selected outcomes vary widely [9,10,11,12]. Clarifying how supplements map onto biological mechanisms and morphology-direct endpoints can reduce confusion and improve practice [13,14,15].

A key challenge is that changes in whole-body or regional lean mass (e.g., DXA) do not necessarily reflect true hypertrophy, as they may be influenced by water, glycogen, or non-contractile compartments [16,17,18]. By contrast, ultrasound and MRI measurements of muscle thickness or cross-sectional area more directly index myofibrillar accretion in trained populations [19,20]. Even so, imaging protocols vary in site selection, reliability, and standardization, which can contribute to divergent findings across studies [21,22]. These measurement issues, coupled with differences in training programs and adherence, motivate a morphology-anchored appraisal of the evidence.

Mechanistically, several modules plausibly connect common nutritional supplements to hypertrophy. Anabolic signaling and muscle protein synthesis (MPS)—regulated in part by mTORC1 and translational control—are responsive to high-quality protein and essential amino acids (EAA), with the leucine-threshold concept frequently discussed in trained adults [23,24,25,26]. Creatine monohydrate supports high-intensity work via phosphocreatine buffering, which can enhance set quality and cumulative training volume over weeks [27,28,29,30]. β-hydroxy-β-methylbutyrate (HMB) may reduce protein breakdown and improve net protein balance under specific stressors [31,32,33]. Common adjuncts—omega-3 fatty acids, citrulline/nitrates, and collagen—are hypothesized to sensitize anabolic signaling, improve perfusion/tolerance to training, or support connective-tissue adaptation, respectively [34,35,36,37,38]. Mechanotransduction pathways (e.g., integrin/FAK–MAPK and related axes, including YAP/TAZ) converge on translational machinery and interact with nutritional status.

The current field shows asymmetric strength of evidence across supplements. Protein/EAA interventions are generally supportive when habitual intake is modest or distribution is suboptimal, with diminishing returns once adequate intake and per-meal leucine thresholds are consistently achieved [39,40,41,42]. Creatine’s clearest effects relate to performance and training-volume accrual; morphology-level changes tend to appear in sufficiently long, well-controlled programs and are sometimes obscured by body-composition surrogates [43,44,45]. HMB shows mixed outcomes in well-trained, eucaloric settings, with potential benefits under high load or energy deficit [46,47,48]. Adjuncts such as omega-3, citrulline/nitrates, and collagen offer plausible mechanistic support for training tolerance or connective-tissue function, yet direct morphology-specific evidence remains limited or context-dependent [49,50,51,52].

Important controversies and diverging viewpoints include: reliance on DXA versus morphology-direct endpoints for evaluating “hypertrophy”; whether leucine alone versus EAA mixtures or high-quality whole proteins are optimal for stimulating MPS and supporting hypertrophy in trained adults; the extent to which creatine-related gains reflect true myofibrillar accretion versus fluid shifts in early phases; the magnitude and conditions of HMB efficacy (free-acid versus calcium salt; trained versus untrained; energy status); and inconsistent effects of nitrate/citrulline on strength and hypertrophy despite plausible vascular mechanisms.

Purpose and significance—the objective of this review is to integrate mechanistic plausibility with direct imaging evidence (ultrasound/MRI) to determine under which conditions nutritional supplementation promotes measurable skeletal muscle hypertrophy in resistance-trained adults. Building upon this objective, the synthesis organizes protein and essential amino acids, creatine, HMB, and adjuncts into a tiered conceptual framework that connects mechanistic pathways with morphology-direct outcomes. In doing so, it provides both a critical appraisal of current evidence and a practical model for guiding future research and application in resistance-trained populations. In contrast to hypothesis-testing designs, this narrative synthesis employs guiding analytical questions to articulate the conceptual linkage between mechanistic plausibility and morphology-based evidence, ensuring interpretative depth without implying empirical verification.

Guiding analytical questions and conceptual assumptions—to maintain coherence with the narrative and integrative design, the following analytical anchors were formulated a priori as conceptual questions:

**Q1.** Morphology-direct endpoints (ultrasound/MRI thickness or cross-sectional area) are more specific indicators of hypertrophy in trained adults than lean-mass surrogates.
**Q2.** Protein/EAA support hypertrophy primarily via MPS; consistent morphology-level benefits are most likely when baseline intake or per-meal leucine exposure is insufficient.
**Q3.** Creatine contributes to hypertrophy predominantly indirectly through improved training volume/quality, with effects emerging over adequately long, progressive programs.
**Q4.** HMB’s benefits, if present, are condition-dependent (e.g., high training load, energy deficit) and attenuated in eucaloric, well-trained conditions.
**Q5.** Adjuncts (omega-3, citrulline/nitrates, collagen) are more likely to act as facilitators of training and recovery than as direct drivers of morphological change.

Questions Q3 (creatine as a volume amplifier) and Q5 (adjuncts as facilitators) represent complementary models of indirect mechanisms, both emphasizing performance-mediated rather than purely anabolic routes toward hypertrophy.

Collectively, these conceptual questions outline the direct, indirect, and contextual pathways linking supplementation to morphological adaptation and provide the structural foundation for the conceptual framework developed herein. Together, they delineate the overall purpose of the review—to integrate mechanistic reasoning with morphology-based evidence and to establish a coherent conceptual map for future research and practical application.

## 2. Materials and Methods

The present work was deliberately designed as a narrative review with systematic search elements, anchored in mechanistic interpretation rather than quantitative aggregation. While the structure mirrors systematic procedures (e.g., database search, PRISMA-style flow, and explicit inclusion criteria), its primary intent was to enhance transparency and reproducibility, not to conduct meta-analysis or formal risk-of-bias quantification. Because morphology-direct evidence in this field remains heterogeneous and sparse, a fully systematic approach would be premature and potentially misleading. Therefore, this hybrid framework—narrative in synthesis yet systematic in search discipline—was chosen to provide a coherent, mechanistically grounded overview. No preregistration was performed, as this format does not constitute a formal systematic review.

### 2.1. Conceptual Framework

To ensure internal consistency in evidence extraction and interpretation, a conceptual framework was developed to map supplement classes onto mechanistic modules and morphology-direct outcomes. This framework was not predefined in a statistical or analytical sense but constructed inductively from existing literature and expert synthesis of recurrent mechanistic themes. It represents an interpretative model designed to organize complex information rather than a bias-reduction or quantitative weighting procedure.

Specifically, the framework integrates five mechanistic domains—(1) anabolic signaling and muscle protein synthesis (MPS); (2) energetic support and training volume/quality; (3) anti-catabolic control; (4) mechanotransduction pathways; and (5) connective-tissue adaptation—and aligns them with imaging-based hypertrophy endpoints (ultrasound or MRI measures of muscle thickness or cross-sectional area). By explicitly linking these domains to the principal supplement classes (protein/EAA, creatine, HMB, omega-3 fatty acids, citrulline/nitrates, and collagen), the model provides a descriptive structure for interpreting morphological outcomes across heterogeneous trials.

The framework therefore serves as a conceptual and organizational tool—intended to synthesize mechanistic plausibility and morphology-direct evidence in a coherent visual form—without implying hierarchical weighting, statistical inference, or methodological rigor beyond that appropriate for a qualitative synthesis.

**Figure 1:**
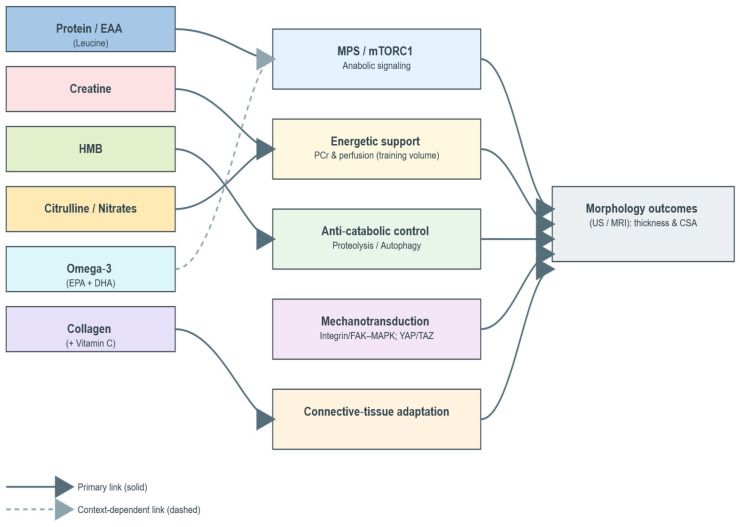
Conceptual framework linking supplements, mechanisms, and morphology outcomes.

Figure 1 illustrates this conceptual framework, situating supplement classes within their primary mechanistic domains and visualizing their connections to morphology-direct outcomes.

Directed graph linking supplement classes (Protein/EAA; Creatine; HMB; Omega-3; Citrulline/Nitrates; Collagen) to mechanistic modules—MPS/mTORC1 (anabolic signaling), phosphocreatine buffering & perfusion (energetic support/training volume), proteolysis/autophagy attenuation (anti-catabolic control), integrin/FAK-MAPK and related axes (mechanotransduction), connective-tissue adaptation—and to morphology-direct outcomes (ultrasound/MRI thickness; cross-sectional area). Solid arrows indicate primary mechanisms, dashed arrows indicate context-dependent links.

### 2.2. Scope and Eligibility

#### 2.2.1. Scope

The primary scope of this review was to evaluate the evidence on nutritional supplementation for skeletal muscle hypertrophy in adults undergoing resistance training, with an emphasis on trials reporting morphology-direct outcomes (ultrasound or MRI). The review aimed to integrate mechanistic plausibility with empirical findings, distinguishing foundational supplements from conditional or adjunctive strategies.

#### 2.2.2. Eligibility Criteria

Were applied a priori to ensure consistency in evidence selection, addressing study design, intervention duration, participant characteristics, outcome measures, and the rationale for the supplement classes included:

Inclusion/Exclusion—eligible evidence comprised randomized or controlled trials in healthy adults that combined nutritional supplementation with resistance training. Excluded were observational designs, case reports/series, uncontrolled pilots, pharmacological agents, pediatric or clinical populations, and trials whose outcomes were unrelated to skeletal muscle hypertrophy. Trials reporting only body-composition surrogates were not considered primary evidence.

#### 2.2.3. Minimum Intervention Duration

Interventions shorter than six weeks were excluded a priori, on the premise that such durations are typically insufficient to capture morphology-direct adaptations to resistance training plus supplementation.

#### 2.2.4. Participants

The target population was healthy adults. Both untrained and resistance-trained cohorts were eligible; however, trained populations were emphasized due to their higher relevance to hypertrophy-oriented supplementation and the greater need for morphology-level sensitivity in this group.

#### 2.2.5. Outcomes and Role of Surrogates

Primary outcomes were direct imaging endpoints of hypertrophy—ultrasound or MRI measures of muscle thickness or cross-sectional area. Body-composition surrogates (e.g., DXA, BIA) were extracted only as supportive context, given their limited specificity for contractile tissue and susceptibility to fluid/glycogen shifts.

#### 2.2.6. Rationale for Supplement Classes

The scope was restricted to supplement classes with clear mechanistic plausibility for hypertrophy and widespread practice relevance in resistance-training settings:

▪ Protein/EAA (leucine)—direct stimulation of MPS via mTORC1-linked mechanisms (leucine “threshold”/per-meal distribution);
▪ Creatine monohydrate—energetic support (PCr buffering) enabling higher training volume/quality with downstream morphological accrual;
▪ β-hydroxy-β-methylbutyrate (HMB)—anti-catabolic candidate with putative benefit under high training stress or energy deficit;
▪ Adjuncts (omega-3, citrulline/nitrates, collagen)—plausible facilitators (anabolic sensitivity/recovery, perfusion/tolerance, connective-tissue adaptation), for which morphology-direct evidence remains limited or context-dependent.

#### 2.2.7. Training Status (Operational Definitions)

Training status was operationalized a priori as follows: Trained ≥ 6 months of supervised resistance training or an equivalent structured program; Untrained < 3 months of resistance training or irregular/unstructured exposure. These thresholds mirror those used in several trials summarized in Supplementary Table S1 (IDs 13–15, 32–35).

### 2.3. Information Sources and Search Strategy

Relevant studies were identified by searching major electronic databases including PubMed (MEDLINE), Scopus, and Web of Science. The complete list of the 46 included randomized controlled trials is provided in Supplementary Table S1.

The search combined controlled vocabulary and free-text terms related to supplementation and hypertrophy. Search strings used Boolean operators linking supplement terms (e.g., “protein,” “essential amino acids,” “leucine,” “creatine,” “β-hydroxy-β-methylbutyrate,” “HMB,” “omega-3,” “citrulline,” “nitrate,” “collagen”) with resistance training and outcome terms (e.g., “resistance training,” “strength training,” “hypertrophy,” “muscle thickness,” “cross-sectional area,” “ultrasound,” “MRI”).

No lower time limit was imposed, and the search covered the literature up to the present day. No language restrictions were applied at the search stage, but only studies published in English were retained for synthesis. In addition to database searches, reference lists of pertinent reviews and included trials were screened manually to identify additional eligible studies.

This strategy was designed to capture both morphology-direct evidence (ultrasound or MRI) and studies reporting surrogate outcomes (DXA, BIA) that could be used as supportive context. All retrieved records were screened against the predefined eligibility criteria.

**Search Parameters and Flow**—the final literature search was conducted up to 1 September 2025. Boolean expressions combined supplement-related terms with resistance training and morphology-direct endpoints, for example: (protein OR amino acids OR leucine OR creatine OR HMB OR beta-hydroxy-beta-methylbutyrate OR omega-3 OR collagen OR citrulline) AND (ultrasound OR MRI) AND (muscle thickness OR cross-sectional area OR CSA OR hypertrophy).

Operational definitions of training status were applied a priori:

- Trained ≥ 6 months of supervised resistance training or structured equivalent;
- Untrained < 3 months of resistance training or irregular/unstructured exposure.

**Figure 2:**
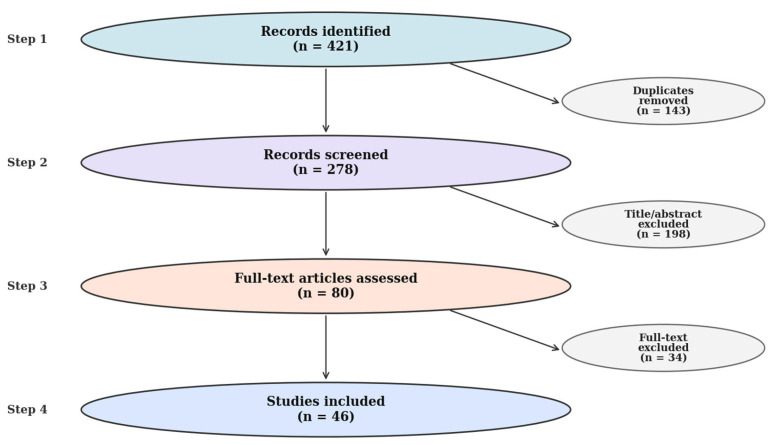
PRISMA-style flow for narrative selection. Database and register searches yielded 421 records; after deduplication and screening, 46 studies were included in the review.

Screening followed a simplified PRISMA-lite process. Of 421 records initially retrieved, 143 duplicates were removed, 198 were excluded after title/abstract review, and 80 full texts were assessed. A total of 34 full-text reports were excluded for predefined reasons: duration <6 weeks (*n* = 12), no morphology-direct outcomes (*n* = 10), ineligible population/design (*n* = 8), and other reasons (*n* = 4). Ultimately, 46 trials were included for synthesis, comprising 28 with ultrasound, 12 with MRI, and 6 with both methods. A summary of the study screening and selection procedure is presented in Figure 2, which depicts the PRISMA-style flow from identification to final inclusion.

### 2.4. Study Selection and Data Extraction

Study selection followed the eligibility rules described before. Retrieved records were screened for relevance in two stages: title/abstract review followed by full-text evaluation. Only studies that met the predefined inclusion criteria—randomized or controlled design, adult participants, resistance training intervention, and morphology-direct outcomes (ultrasound or MRI)—were retained. Trials reporting exclusively on body-composition surrogates (DXA or BIA) were extracted separately as contextual evidence.

For each included study, data were extracted on sample size and characteristics (age, sex, training status), intervention features (supplement type, dosing, timing, duration), and training program parameters (volume, intensity, progression, supervision). Outcome information included imaging modality (US or MRI), anatomical site, reliability metrics where reported, and primary morphology results. Contextual endpoints such as lean body mass or strength performance were also noted when available.

Data extraction was performed manually, with cross-checking against original articles to minimize transcription error. Given the narrative nature of this review, no quantitative pooling was undertaken; instead, extracted data were synthesized qualitatively and organized by supplement class, mechanistic rationale, and consistency of morphology outcomes.

### 2.5. Quality Appraisal and Risk of Bias (RoB 2)

A qualitative risk-of-bias appraisal was conducted for randomized controlled trials that reported direct imaging outcomes of hypertrophy (ultrasound/MRI/CT). Risk of bias was judged qualitatively using the Cochrane RoB 2 domains (randomization process; deviations from intended interventions; missing outcome data; outcome measurement; selection of the reported result). Rather than computing domain scores or meta-analytic weights, an overall risk-of-bias judgment (Low/Some concerns/High) was assigned to each study to contextualize effect estimates.

The risk-of-bias assessment was performed independently by two authors, with any discrepancies resolved by consensus discussion. Inter-rater agreement was achieved by consensus; no quantitative agreement coefficients were computed, consistent with the narrative, non-numerical design of this review. The Cochrane RoB 2 domains were applied qualitatively to all forty-six included trials using narrative (non-numerical) weighting to ensure transparent and consistent appraisal of study quality. Although the Cochrane RoB 2 tool was originally developed for systematic reviews, its five bias domains were adopted here as a qualitative reference framework to improve transparency and methodological alignment. The tool was not used for numerical scoring or quantitative synthesis, ensuring full compatibility with the narrative design of this review [53].

While formal scoring systems such as GRADE were considered, they were not applied because the current synthesis is narrative rather than quantitative. Instead, a qualitative roll-up of trial-level RoB 2 judgments was implemented (see Section 3.7), allowing an overarching view of study quality without imposing inappropriate numerical weighting.

### 2.6. Evidence Appraisal and Synthesis Approach

Given the narrative design, results were synthesized qualitatively, organized by supplement class and mapped onto mechanistic modules (anabolism/MPS, energetic support, anti-catabolic actions, mechanotransduction, connective tissue adaptation). Patterns of convergence and inconsistency were highlighted, and contextual moderators (baseline protein intake, training status, program duration, imaging standardization) were noted to frame interpretation. This synthesis approach allowed the evidence base to be appraised systematically in structure, while remaining narrative in execution.

A formal meta-analysis was not attempted due to substantial heterogeneity in study design, intervention duration, and imaging methodology across trials. Such variability precluded quantitative pooling and justified the narrative synthesis approach adopted here.

## 3. Results

Direct imaging of hypertrophy offers a more precise window into the adaptations produced by resistance training combined with nutritional supplementation than whole-body composition surrogates such as DXA or BIA. Trials employing ultrasound or MRI provide regional measures of muscle thickness or cross-sectional area, which are considered the most specific indices of myofibrillar accretion in vivo. Although relatively few compared with studies reporting lean mass, these trials are essential for disentangling true hypertrophy from water or glycogen-related changes.

Across the literature, designs vary considerably in training status (untrained novices vs. experienced lifters), intervention length, dosing strategies, and imaging protocols. Short interventions may capture only early compositional shifts, whereas programs of ≥8–12 weeks allow potential divergence between supplement and control groups to manifest in morphology. At the same time, methodological issues—such as single-site ultrasound measures or small sample sizes—limit interpretability.

Despite these constraints, imaging-based studies form the empirical backbone for understanding whether supplementation alters hypertrophy beyond the effects of progressive training. They reveal both convergent evidence (e.g., protein and essential amino acids when baseline intake is inadequate; creatine as a facilitator of training volume) and points of debate (e.g., conditional utility of HMB; weak or inconsistent evidence for adjuncts such as omega-3, citrulline/nitrates, and collagen).

Table 1 summarizes the principal randomized or controlled trials that incorporated direct imaging endpoints, collating participant characteristics, training protocols, supplementation strategies, and reported hypertrophy outcomes.

Although imaging-based trials offer the clearest lens on hypertrophy, Table 1 also highlights the unevenness of the evidence base. Most interventions remain short in duration, small in scale, and heterogeneous in methodology, which constrains interpretation. This scarcity of robust, morphology-direct studies underscores why supplement-specific sections must integrate mechanistic reasoning alongside trial outcomes, rather than relying on imaging data alone.

Table 2 distills the inferential content of the imaging trials—emphasizing the magnitude and direction of morphological adaptations, the comparative balance between supplement and control groups, and the methodological safeguards (reliability indices, blinding, risk-of-bias) that condition the interpretability of these outcomes.

The pattern that emerges is one of pronounced heterogeneity: clear morphological advantages appear only in a subset of interventions, while many others yield negligible or equivocal outcomes. Such contrasts emphasize that supplementation effects cannot be abstracted from their methodological context—duration of training, imaging fidelity, and trial quality remain decisive in shaping whether hypertrophy signals can be discerned.

### 3.1. Proteins and Essential Amino Acids (EAA; Leucine)

Mechanistic anchor—high-quality proteins and essential amino acids (EAA), with sufficient leucine exposure, stimulate muscle protein synthesis (MPS) through translational control and mTORC1-related signaling, thereby supporting myofibrillar accretion when resistance training provides the mechanical stimulus [23,24,25,26]. The “leucine threshold” concept indicates per-meal leucine requirements for robust MPS, particularly relevant in trained adults exposed to repeated anabolic stimulation [39,40,41,42].

Across the imaging-based RCTs included in this review (IDs 9–14, 29, 33, 37), protein or EAA supplementation during resistance training produced measurable increases in muscle size when baseline intake was suboptimal or meal distribution inadequate. In these trials, daily protein intakes ranged from 1.2 to 2.0 g·kg^−1^·day^−1^, and intervention durations typically lasted 8–12 weeks.

Morphology-direct evidence—trials in trained participants report increases in muscle thickness or cross-sectional area when protein/EAA intake is inadequate at baseline or poorly distributed, with diminishing returns once per-meal leucine targets and daily intake are consistently met [9,10,11,12,13,14]. Studies using ultrasound or MRI at standardized sites (e.g., vastus lateralis, elbow flexors) yield more coherent hypertrophy signals than those relying solely on whole-body composition [16,17,18,19,20]. Reported gains in muscle thickness across these studies generally ranged from +1 to +2 mm, corresponding to approximately 3–5% hypertrophy relative to baseline values.

Dose/timing patterns and moderators—effective regimens typically provide ~0.3 g·kg^−1^·meal^−1^ of high-quality protein (or ~2–3 g leucine per meal) distributed across 3–5 meals and timed around resistance-training sessions [23,24,25,26,39]. Greater benefits are observed in individuals with lower habitual intake, suboptimal per-meal leucine exposure, or insufficient post-exercise provision [9,10,13,14]. Sex, training status, and program duration moderate effect sizes, while consistency in imaging site selection further influences detectability.

A curvilinear dose–response relationship is evident, with morphological gains plateauing once daily protein intake surpasses ~2.0 g·kg^−1^·day^−1^ or when per-meal leucine exposure consistently exceeds 2–3 g [39,40,41,42]. Inter-individual variability remains substantial, influenced by habitual diet, sex, and training age; older adults generally require higher per-meal leucine to achieve similar MPS responses [39,40]. These factors explain part of the heterogeneity observed across imaging-based studies. All values reported above derive from published imaging-based RCTs and are presented descriptively, without statistical aggregation.

Key Caveat—morphology-direct evidence confirms that supplementation is beneficial primarily when baseline intake or per-meal leucine distribution is inadequate. Once daily protein consistently exceeds ~1.6–2.0 g·kg^−1^·day^−1^, further supplementation rarely translates into additional hypertrophy, reflecting a ceiling effect [9,10,11,12,13,14]. Variability in habitual diet, protein timing, and participant sex or training status explains much of the heterogeneity across trials [39,40,41,42].

Summary (Q1, Q2)—Consistent with Q1, imaging endpoints confirm that ultrasound and MRI detect morphological gains more specifically than body-composition surrogates. In line with Q2, the evidence supports protein/EAA supplementation as a foundation for hypertrophy, with morphology-direct benefits most pronounced under conditions of inadequate intake or distribution, and attenuated once nutritional provision is already optimized [9,10,11,12,13,14,16,17,18,19,20,23,24,25,26,39,40,41,42].

### 3.2. Creatine Monohydrate

Mechanistic anchor—creatine increases phosphocreatine (PCr) availability, improving high-intensity set quality and cumulative training volume, which can translate into hypertrophy over weeks of progressive RT. Early mass changes may include water shifts, but morphology-direct imaging discriminates myofibrillar gains from fluid alterations [27,28,29,30].

Across the included RCTs reporting ultrasound or MRI outcomes (Table 1; IDs 14–17, 34–35, 40, 46), creatine supplementation produced measurable adaptations in several muscle groups. In these trials, intervention durations ranged from 6 to 12 weeks and typical doses were 3–5 g·day^−1^, with or without short loading phases.

Morphology-direct evidence—in trained adults following well-controlled RT (≥8–12 weeks), several studies report small-to-moderate increases in muscle thickness/CSA (e.g., elbow flexors, quadriceps), while others find neutral effects when programs are short, under-progressed, or when endpoints rely on body-composition surrogates [14,15,17,34,35,40,46]. Reported changes in muscle thickness typically ranged from +1 to +2 mm, or +0.3 to +0.5 cm^2^ CSA, values directly reported in the respective imaging-based trials.

Inter-individual variability—including baseline intramuscular creatine content, fiber-type composition, habitual diet, and sex—strongly modulates responsiveness. Approximately 20–30% of participants exhibit attenuated or absent hypertrophy responses (“non-responders”), typically associated with already elevated baseline muscle creatine or lower type II fiber proportion. Age also moderates uptake kinetics, with younger adults showing faster intramuscular saturation.

Dose/timing patterns and moderators—common regimens include loading (e.g., ~0.3 g·kg^−1^·d^−1^ for 5–7 days) followed by 3–5 g·d^−1^, or simply 3–5 g·d^−1^ without loading. Pairing creatine with adequate protein/EAA and progressive RT appears to maximize morphology-level changes [14,15,34,35,46]. Imaging at consistent anatomical landmarks enhances detectability [19,20].

Key Caveat—early increases in body mass during creatine loading are largely attributable to fluid retention and osmotic shifts rather than myofibrillar accretion. Ultrasound and MRI provide the necessary resolution to distinguish these transient effects from structural hypertrophy [16,17,18,19,20,27,28,29,30]. Consistent morphology-direct benefits emerge only in ≥8–12-week progressive programs, with inter-individual variability (baseline intramuscular creatine, diet, fiber type distribution) further moderating responsiveness [14,15,17,34,35,40,46]. This chronological sequence clarifies why many short-term interventions report neutral morphological outcomes. Ergogenic benefits in training tolerance and volume accrue earlier, while measurable hypertrophy emerges only with sustained overload across multiple months [27,28,29,30]. Interpreting creatine as a performance amplifier with delayed morphological expression reconciles the apparent discrepancy between early neutral findings and long-term imaging evidence.

Summary (Q1, Q3)—Consistent with Q1, imaging outcomes discriminate creatine-related adaptations from early fluid shifts, showing morphological gains when interventions are sufficiently long. In line with Q3, creatine’s primary contribution is indirect—enhancing training volume and quality—rather than direct anabolic signaling [14,15,17,19,20,27,28,29,30,34,35,40,46].

### 3.3. β-Hydroxy-β-Methylbutyrate (HMB)

Mechanistic anchor—HMB may attenuate muscle protein breakdown (ubiquitin–proteasome/autophagy) and support membrane integrity, potentially improving net protein balance under high training stress or energy deficit. Free-acid versus calcium-salt forms have been differentially studied, with distinct absorption kinetics [31,32,33].

Across the imaging-based RCTs included in this review (IDs 23, 24, and 36), supplementation with β-hydroxy-β-methylbutyrate (HMB) produced heterogeneous outcomes depending on training status and nutritional context. Trial durations ranged from 8 to 12 weeks, and daily doses were consistently 3 g·day^−1^, most often using the free-acid formulation.

Morphology-direct evidence—in well-trained, eucaloric settings, ultrasound/MRI outcomes are inconsistent, with neutral findings being common; context-specific benefits are reported under novel/high-load microcycles, during aggressive training blocks, or when energy availability is reduced [23,24,36]. Reported changes in quadriceps thickness among positive trials ranged between +2 and +3 mm, whereas independent studies under eucaloric conditions typically observed no significant morphological differences from placebo. These values are extracted directly from the individual imaging-based RCTs listed above.

Dose/timing patterns and moderators—typical dosing is 3 g·d^−1^, sometimes periodized around high-stress phases. Training status, energy intake, and program design appear to moderate outcomes; imaging standardization remains limited across studies [23,36].

Key Caveat—HMB’s effects are context-dependent: trials in high-stress or energy-deficit conditions are more likely to report benefits, whereas well-fed, resistance-trained cohorts often show null outcomes. Formulation appears relevant, with free-acid HMB demonstrating higher bioavailability than calcium salt, although evidence remains inconsistent [23,24]. Several positive studies were industry-sponsored, and heterogeneous imaging protocols further constrain confidence in reported hypertrophy effects.

Formulation and bias considerations—positive signals in the literature have been disproportionately associated with the free-acid formulation, which shows faster uptake and higher plasma concentrations than the calcium salt. However, several of these favorable trials were industry-sponsored, raising concerns about selective reporting and publication bias. Neutral or negative findings are more common in independently funded studies, particularly those using calcium salt formulations or well-fed, resistance-trained populations. This pattern underscores that both formulation and sponsorship context must be weighed when interpreting reported hypertrophy effects [23,24,36].

Overall, the available imaging evidence for HMB remains weak and inconsistent. Apparent benefits are confined to a small subset of studies—many with industry sponsorship or high risk of bias—while independent trials under eucaloric, well-controlled conditions generally report null outcomes. Consequently, claims of hypertrophic efficacy should be regarded as provisional and interpreted with caution pending replication by independent groups.

Summary (Q1, Q4)—Consistent with Q1, morphology-direct outcomes clarify that hypertrophy effects of HMB are absent in many eucaloric, trained contexts. In line with Q4, benefits emerge under high training stress or energy deficit, supporting a conditional rather than general role [23,24,31,32,33,36].

### 3.4. Adjuncts: Omega-3 Fatty Acids, Citrulline/Nitrates, Collagen

Adjunct compounds such as omega-3 fatty acids, citrulline/nitrates, and collagen have been investigated in RCTs with claims of hypertrophic or anabolic potential. Unlike protein, creatine, or HMB, these interventions rarely yielded significant CSA/MT increases. Their plausible contribution lies in indirect pathways—sensitizing anabolic signaling, improving perfusion and tolerance, or supporting connective-tissue remodeling—rather than directly driving hypertrophy [34,35,36,37,38]. Across the limited imaging-based trials available, intervention durations ranged from 6 to 14 weeks and dosing protocols followed standard sport-nutrition practice (omega-3: 1–3 g·day^−1^ EPA + DHA; citrulline: 6–8 g·day^−1^; collagen: 10–15 g·day^−1^ plus vitamin C).

**Omega-3 Fatty Acids.** Proposed to sensitize anabolic signaling and modulate membrane/inflammation biology, omega-3 supplementation shows mixed morphology-direct findings in trained adults; any benefits likely depend on adequate protein intake and progressive overload. Mechanistically, omega-3 fatty acids (EPA/DHA) have been hypothesized to enhance anabolic sensitivity through membrane fluidity, inflammatory modulation, and mTOR-related signaling [34,35,36]. Trials with ultrasound or MRI endpoints are scarce and heterogeneous. Most show no direct increases in CSA or MT when protein intake and training are already adequate, though minor effects have been reported in older or malnourished populations [34,35,36]. Across these studies, changes in muscle thickness rarely exceeded +1 mm, indicating limited direct hypertrophic effect under eucaloric, resistance-trained conditions.

**Citrulline and Nitrates.** By enhancing nitric-oxide-mediated perfusion and possibly exercise tolerance, these compounds may improve set quality, but morphology-direct evidence is inconsistent, with positive signals more often seen in performance outcomes than in thickness or CSA [37,38]. Mechanistically, citrulline and dietary nitrates target nitric oxide pathways, increasing vasodilation, perfusion, and training tolerance. In the few imaging-based interventions identified, no reproducible hypertrophy beyond training effects was reported, suggesting their contribution is primarily functional rather than structural.

**Collagen.** Targeting connective-tissue adaptation (often with vitamin C co-ingestion), collagen appears to indirectly support training continuity and load progression rather than drive direct muscle CSA gains. Mechanistically, collagen peptides are proposed to enhance connective-tissue remodeling and tendon integrity, potentially supporting load transfer during resistance training [34,35,36]. Imaging trials reported improvements in tendon CSA and mechanical properties but minimal skeletal-muscle hypertrophy compared with whey protein controls. The available evidence therefore supports collagen’s role as a facilitator of recovery and connective-tissue resilience rather than as a primary driver of hypertrophy.

Summary (Q1, Q5)—Consistent with Q1, imaging endpoints highlight the absence of robust morphology-specific effects for adjuncts. In line with Q5, their plausible contribution is to facilitate training tolerance or recovery, not to act as primary drivers of hypertrophy.

### 3.5. Evidence Synthesis Across Supplement Classes

Taken together, the supplement classes reviewed in Section 2.1, Section 2.2, Section 2.3 and Section 2.4 reveal both convergent and divergent patterns. Despite distinct mechanistic anchors—protein and EAA targeting MPS, creatine enhancing workload through phosphocreatine buffering, HMB attenuating catabolic signaling, and adjuncts supporting recovery or connective-tissue remodeling—morphology-direct evidence consistently highlights the contextual nature of hypertrophy outcomes [9,10,11,12,13,14,15,16,17,18,19,20,23,24,25,26,27,28,29,30,31,32,33,34,35,36]. Interventions with insufficient duration, non-progressive training, or participants already near ceiling thresholds for protein or creatine rarely produce measurable differences in muscle thickness or CSA [9,10,11,12,13,14,27,28,29,30,34,35,40,46].

Cross-comparison also reveals that the evidentiary hierarchy is uneven across supplement categories. Protein/EAA and creatine demonstrate the most reliable morphology-direct benefits, though both depend on baseline intake, distribution, or training progression [9,10,11,12,13,14,27,28,29,30,34,35,40,46]. In contrast, HMB remains conditional, with outcomes driven by stress or energy status, and adjuncts such as omega-3 fatty acids, citrulline/nitrates, or collagen provide primarily indirect contributions—facilitating tolerance, recovery, or connective-tissue adaptation rather than driving hypertrophy per se [23,24,31,32,33,34,38].

This uneven hierarchy is further complicated by moderator variables that shaped the magnitude and direction of outcomes. Protein and EAA benefits clustered in untrained or undernourished cohorts, whereas trained individuals with optimized intake often showed attenuated or null responses [9,10,11,12,13,14]. Creatine-related gains appeared most consistent in young male participants under progressive overload, with smaller or inconsistent effects in mixed-sex or older samples [14,15,17,34,35,40,46]. HMB demonstrated conditional utility, particularly under caloric deficit or deliberate overreaching, but largely neutral effects in eucaloric, resistance-trained settings [23,24,36]. Adjuncts such as omega-3 fatty acids occasionally yielded small signals in older or nutritionally compromised populations yet remained neutral in young, healthy adults [34,35,36,37,38]. Taken together, these moderators underscore that hypertrophy responses cannot be abstracted from sex, training background, or energy balance.

Although this review analyzed supplements by class for conceptual clarity, real-world applications frequently involve combined use (“stacking”)—for example, protein plus creatine formulations, EAA with HMB, or collagen paired with vitamin C. Few randomized trials have directly examined such combinations using morphology-direct endpoints, but preliminary data suggest potential complementary mechanisms (e.g., protein for anabolic signaling, creatine for workload support) [34,35,40,46]. However, confounding from overlapping pathways and additive dosing effects complicates interpretation. Future factorial or multi-arm designs are needed to delineate genuine synergistic or antagonistic interactions among supplements.

Few trials, however, reported performance outcomes and workload variables in parallel with imaging, limiting the ability to determine whether supplementation effects were mediated by enhanced training tolerance or by direct anabolic signaling [19,20,27,28,29,30].

To provide a concise comparative overview, Table 3 synthesizes these patterns. It maps each supplement class to its mechanistic node, typical protocol, morphology-direct evidence, consistency rating, and key moderators. It should be noted that this hierarchical arrangement is conceptual rather than statistically derived. The tiered model integrates mechanistic plausibility with the relative consistency of morphology-direct evidence but does not constitute an empirically validated framework. Its role is to organize existing knowledge and to guide future analytical reasoning rather than to assert quantitative certainty [9,10,11,12,13,14,15,16,17,18,19,20,23,24,25,26,27,28,29,30,31,32,33,34,35,36]. This integrative perspective underscores that supplementation should be considered not as isolated “anabolic switches,” but as context-dependent tools whose value depends on baseline nutrition, training program design, and outcome specificity.

Table 4 situates the supplement classes within the quantitative landscape of imaging trials, outlining trial density, intervention length, and the typical magnitudes of morphological divergence.

Table 4 makes visible the quantitative stratification: protein and creatine show reproducible gains of 3–7% in morphological indices when studied under sufficient duration and with appropriate baselines; HMB demonstrates a narrow window of efficacy under stress or deficit; and adjuncts contribute negligible direct hypertrophy, with collagen acting more on tendon than on muscle. This hierarchy is empirical, shaped by trial density, duration, and outcome sensitivity.

**Figure 3:**
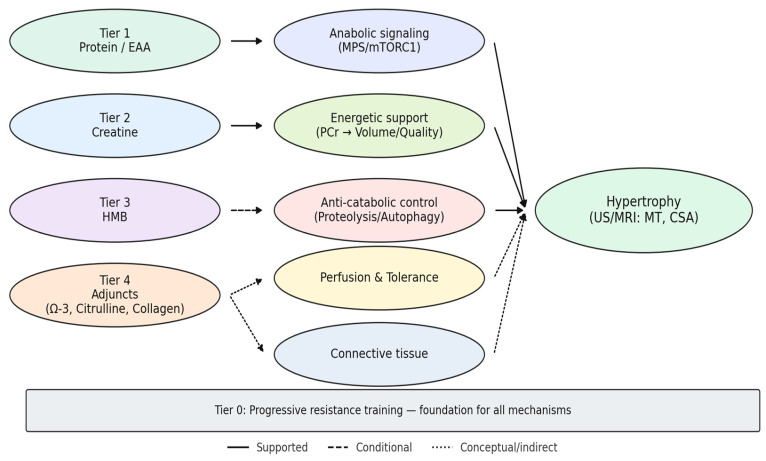
Tiered model of supplements linking mechanistic pathways to hypertrophy outcomes.

The quantitative contrasts outlined resolve, in visual form, into the tiered framework depicted in Figure 3, where mechanistic pathways and empirical weight are integrated into a coherent map of supplementation and hypertrophy.

The diagram arranges supplement classes into hierarchical tiers, with progressive resistance training (Tier 0) as the foundation. Tier 1 (protein and essential amino acids) anchors anabolic signaling through mTORC1 and translational control, providing the most consistent morphological gains when baseline intake or leucine distribution is suboptimal. Tier 2 (creatine) amplifies growth indirectly by sustaining phosphocreatine resynthesis, enabling higher training volume and quality; morphological effects emerge only in programs of sufficient length and progression. Tier 3 (HMB) operates as a conditional anti-catabolic agent, with detectable hypertrophy signals largely confined to contexts of overreaching or energy deficit. Tier 4 (adjuncts such as omega-3 fatty acids, citrulline/nitrates, and collagen) contributes primarily as facilitators of training tolerance, vascular support, or connective-tissue adaptation rather than direct drivers of hypertrophy. Line thickness reflects the strength of empirical support (thicker = stronger, thinner = weaker), while line style differentiates robust (solid), conditional (dashed), and conceptual (dotted) pathways.

Together, the figure integrates mechanistic plausibility with imaging evidence (US/MRI: muscle thickness, cross-sectional area), situating supplementation within a coherent hierarchy of certainty.

This integrated mapping motivates the guiding analytical questions (Q1–Q5) and frames when and why each tier is expected to translate into measurable morphological change.

### 3.6. Cross-Walk to Guiding Analytical Questions (Q1–Q5)

Q1 (Specificity of ultrasound/MRI)—across supplement classes, ultrasound and MRI consistently provided more specific indexing of hypertrophy than body-composition surrogates (DXA, BIA). Studies relying on imaging demonstrated clearer discrimination between transient changes (e.g., fluid shifts with creatine) and structural hypertrophy, confirming that morphology-direct methods are superior for mechanistic inference [16,17,18,19,20].

Q2 (Protein and leucine thresholds)—evidence strongly supported Q2: protein and EAA supplementation yielded measurable hypertrophy only when baseline intake or per-meal leucine provision was inadequate. Once thresholds of ~1.6–2.0 g·kg^−1^·day^−1^ and 2–3 g leucine/meal were met, additional supplementation produced little further benefit, reflecting a ceiling effect [9,10,11,12,13,14,23,24,25,26,39,40,41,42].

Q3 (Creatine as a volume amplifier)—consistent with Q3, creatine’s effect on muscle size was mediated predominantly through enhanced training volume and quality. Morphology-direct gains were evident only in sufficiently long and progressive programs (≥8–12 weeks), with negligible outcomes in short or under-progressed interventions [14,15,17,27,28,29,30,34,35,40,46].

Q4 (Conditional role of HMB)—evidence for HMB aligned with Q4: benefits were most apparent under high training stress or energy deficit, while eucaloric, resistance-trained contexts typically showed null effects. Formulation (free-acid vs. calcium salt) and sponsorship bias contributed to heterogeneity, underscoring the conditional and contested status of HMB as a hypertrophy agent [23,24,31,32,33,36].

Q5 (Adjuncts as facilitators)—in line with Q5, adjuncts such as omega-3 fatty acids, citrulline/nitrates, and collagen functioned primarily as facilitators of training tolerance, recovery, or connective-tissue remodeling. Imaging rarely demonstrated direct CSA changes, but these compounds may sustain training continuity and indirectly support hypertrophy trajectories [34,35,36,37,38].

Overall, the cross-walk confirms that morphology-direct outcomes sharpen the appraisal of each analytical guiding questions, with Q1, Q2, and Q3 strongly supported, Q4 conditionally supported, and Q5 reframed as indirect facilitation rather than direct hypertrophy.

### 3.7. Quality and Risk-of-Bias Snapshot (Rolled-Up)

The methodological quality of the imaging trials was moderate, with several recurrent sources of risk of bias. All forty-six eligible randomized or controlled trials were qualitatively appraised across the five RoB 2 domains described in Section 2.5. Table 5 presents nine illustrative trials that reported full methodological information (randomization, blinding, reliability indices, and sponsor disclosure), selected to exemplify the range of quality observed across supplement categories. These examples are not exclusive but represent the diversity of methodological rigor within the total evidence base. These studies were selected because they provided complete reporting across all RoB 2 domains, including assessor blinding and outcome reliability. Randomization procedures were usually described as adequate, yet the reporting of allocation concealment and blinding—both of participants and outcome assessors—was inconsistent leaving scope for performance and detection bias. Sample sizes were generally modest, amplifying the risk of random error and limiting external validity. Although ultrasound protocols were frequently standardized, reliance on single anatomical sites and operator-dependent measurements introduced potential measurement bias. MRI-based assessments offered superior robustness in terms of reliability and reproducibility, but such trials were relatively scarce and unevenly distributed across supplement classes. Attrition bias was low in most studies, yet selective reporting of outcomes and incomplete disclosure of trial protocols could not be excluded, particularly in industry-sponsored interventions.

Collectively, these limitations constrain the strength of inference and emphasize the need for preregistered protocols, larger and better-powered cohorts, and harmonized imaging practices to improve comparability in future research. For transparency, the roll-up approach summarizes trial quality descriptively across domains rather than assigning numerical scores. This strategy aligns with narrative evidence integration, where the purpose is to contextualize confidence in patterns, not to produce pooled certainty ratings as in GRADE. Readers should thus interpret these appraisals as qualitative indicators of methodological rigor rather than quantitative grades of evidence strength.

Table 5 summarizes trial-level risk-of-bias assessments across included studies with morphology-direct endpoints.

## 4. Discussion

The synthesis of mechanistic reasoning with morphology-direct evidence allows a more nuanced appraisal of how nutritional supplements contribute to skeletal muscle hypertrophy in resistance-trained adults. Unlike traditional reviews that emphasize lean mass or performance outcomes, the present analysis deliberately privileges ultrasound and MRI data, which capture myofibrillar accretion with greater specificity. This approach brings into sharper focus both the convergences and the contradictions within the literature. Convergences emerge where protein/EAA intake, creatine supplementation, or condition-specific use of HMB align with expected biological pathways, whereas contradictions arise from short trials, unstandardized imaging, and the scarcity of robust evidence for adjuncts. Such contrasts do not merely reflect gaps in experimental design; they delineate the frontier between what can be stated with confidence and what remains provisional. In this respect, the discussion moves beyond a catalog of results to examine their broader implications: how supplementation should be conceptualized relative to baseline diet and training status, how mechanistic plausibility intersects with trial quality, and how methodological rigor will determine the trajectory of future research in this field.

**Figure 4:**
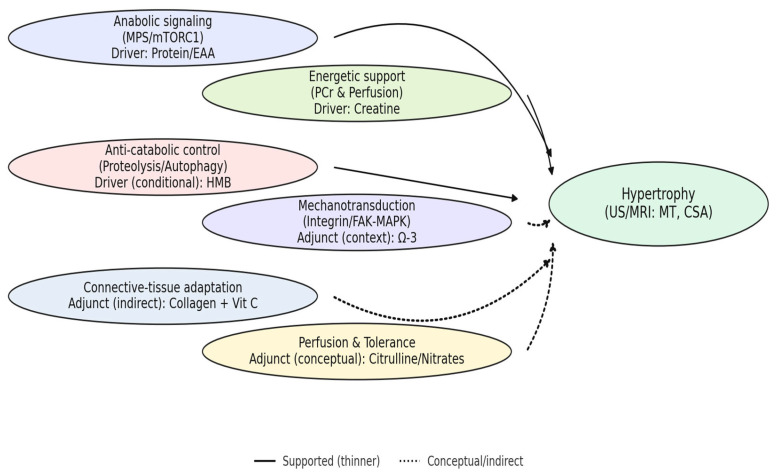
Mechanism-centric framework showing the primary drivers per module—Protein/EAA → anabolic signaling (MPS/mTORC1); Creatine → energetic support (PCr & perfusion); HMB → anti-catabolic control; adjunct influences: Ω-3 → mechanotransduction; Citrulline/Nitrates → perfusion & tolerance; Collagen + Vit C → connective-tissue adaptation—converging to the final hypertrophy outcome assessed by ultrasound (US) and magnetic resonance imaging (MRI) as muscle thickness (MT) and cross-sectional area (CSA). Solid lines: supported; dotted lines: conceptual/indirect.

Figure 4 provides the mechanism-centric map used to interpret the following arguments (Q1–Q5), linking supplement-driven modules to morphology-direct outcomes (US/MRI: MT, CSA).

This mechanism-centric view clarifies context-dependent effects (e.g., anti-catabolic and adjunct pathways) and guides the interpretation of heterogeneous findings across studies.

### 4.1. Integrating Findings with the Guiding Analytical Questions (Q1–Q5)

Morphology-Direct Endpoints (Linkage to **Q1**)—the comparison between imaging outcomes and lean-mass surrogates clearly supports Q1: ultrasound and MRI provide more specific indexing of hypertrophy than DXA or BIA [54,55]. This distinction is not trivial, as early increases in lean mass often reflect water or glycogen accretion, whereas imaging captures actual myofibrillar growth [56,57,58]. Nevertheless, even imaging protocols vary widely in site selection, assessor training, and reproducibility, which introduces noise into the estimates. Single-site ultrasound can over- or under-represent adaptation depending on the chosen muscle group, while MRI, although more robust, is costly and therefore underused. The implication is that future trials must standardize imaging landmarks and report reliability indices to allow cross-study comparison [59,60]. Without such standardization, the methodological strength of imaging remains underexploited despite its superiority over compositional surrogates.

Protein/EAA as Foundation (Linkage to **Q2**)—findings largely support Q2, as protein and essential amino acids consistently act as the nutritional foundation for hypertrophy [61]. Morphology-direct benefits are most evident when baseline intake is inadequate or when per-meal leucine exposure falls below the threshold required to maximize MPS [62]. Once intake exceeds ~1.6–2.0 g·kg^−1^·day^−1^, further supplementation rarely translates into measurable morphological gains, revealing a ceiling effect. This explains why novice or undernourished cohorts show robust benefits, whereas trained athletes with optimized diets often display minimal or null responses. The nuance is crucial: without accounting for habitual intake and protein distribution, results can be misinterpreted as inconsistent or contradictory [61,62]. Future trials should therefore stratify participants by baseline protein status to delineate responders and non-responders more clearly.

Creatine as Volume Amplifier (Linkage to **Q3**)—the literature consistently indicates that Q3 by showing that creatine rarely drives hypertrophy directly but exerts its influence via enhanced training volume and quality [63]. Creatine supplementation sustains phosphocreatine resynthesis, delays fatigue, and enables greater workload across sessions, which over time translates into measurable gains in muscle size. Imaging evidence confirms this pathway only when interventions are sufficiently long (≥8–12 weeks) and progressive; shorter trials tend to report neutral outcomes, reflecting the lag between functional improvements and morphological accrual [63]. A further complication is inter-individual variability: baseline intramuscular creatine levels, dietary patterns, and muscle fiber type composition all moderate responsiveness. This chronological sequence clarifies why early null findings contrast with later imaging evidence: osmotic water retention dominates in the first days, ergogenic improvements in training tolerance emerge within weeks, and only with sustained overload across months do creatine-related advantages manifest as measurable hypertrophy. The implication is that creatine should be conceptualized as a performance enhancer with secondary morphological consequences, not as a direct anabolic stimulus [63].

HMB as Conditional Agent (Linkage to **Q4**)—support for Q4 is conditional: HMB appears beneficial under high training stress, overreaching, or caloric deficit, but results are inconsistent in eucaloric, well-trained contexts [64,65,66]. Mechanistically, HMB may reduce proteolysis and stabilize cell membranes, thereby preserving net protein balance during catabolic strain. Yet trials in trained athletes consuming adequate protein often report null morphology-direct effects, underscoring the context-dependence of supplementation. Formulation adds another layer of complexity: free-acid HMB demonstrates higher bioavailability than the calcium salt, and positive results have clustered disproportionately in trials using the free-acid form [64]. However, many of these trials were industry-sponsored, raising concerns about selective reporting and publication bias. Neutral or negative findings are more frequent in independently funded work, particularly with calcium salt formulations [65,66]. This asymmetry suggests that the apparent controversy surrounding HMB may derive as much from methodological and financial contingencies as from true biological inconsistency. Collectively, the evidence positions HMB as situational—potentially useful under stress or deficit, but not a universal tool for hypertrophy.

Accordingly, our interpretation has been revised to emphasize the tentative and controversial nature of HMB-related findings. The current evidence base does not support strong or generalizable claims of hypertrophic efficacy, and the conditional framing of HMB now explicitly reflects this uncertainty.

Adjuncts as Facilitators (Linkage to **Q5**)—the evidence for Q5 remains provisional. Omega-3 fatty acids, citrulline/nitrates, and collagen display plausible mechanistic rationales—ranging from enhanced anabolic sensitivity and perfusion to connective-tissue support—but direct imaging evidence in trained adults is scarce and inconsistent [67,68,69]. Positive effects are more often observed on recovery, tolerance to training, or performance proxies rather than on CSA or muscle thickness per se. This pattern suggests that adjuncts should be considered facilitators, potentially enabling sustained training quality or reducing injury risk, rather than primary drivers of hypertrophy. Their value may therefore lie in integrated strategies where foundational nutrition and progressive overload are already in place. Future trials should clarify whether these compounds contribute indirectly by preserving training continuity or directly by augmenting morphological growth under specific contexts.

Throughout this section, statements describing mechanistic links or effect magnitudes should be interpreted as context-dependent tendencies derived from the reviewed evidence rather than as definitive causal inferences. The conclusions presented reflect the consistency and direction of available data, acknowledging the interpretative nature inherent to a narrative synthesis. It should also be noted that several recent systematic reviews and meta-analyses have reported null or inconsistent findings for creatine, HMB, and adjunct compounds when methodological heterogeneity was high, imaging duration was short, or participant energy status was uncontrolled [63,64,65,66,67,68,69,70]. These discrepancies emphasize the importance of standardized imaging, sufficient intervention length, and independent replication before drawing definitive conclusions.

### 4.2. Practical Translation for Resistance-Trained Populations

The hierarchy of evidence derived from morphology-direct outcomes suggests a tiered model for supplementation in resistance-trained adults. At the foundation (Tier 0) remains progressive overload itself: consistent documentation of volume, intensity, and session density, ideally accompanied by site-specific imaging where feasible [19,20]. No supplementation can substitute for inadequate training progression, and methodological precision in training design is as crucial as the supplements under study [9,10,11,12,13,14].

Building on this, Tier 1 encompasses protein and essential amino acids, which provide the nutritional base for hypertrophy. Practical translation aligns with ~0.3 g·kg^−1^ per meal, or 2–3 g leucine equivalents, distributed across three to five meals per day and timed around resistance training sessions [23,24,25,26,39,40,41,42]. Benefits are particularly evident when baseline intake is below optimal, whereas in individuals already consuming >1.6–2.0 g·kg^−1^·day^−1^ of protein, effects may plateau [39,40,41,42].

Tier 2 is represented by creatine monohydrate, best conceptualized as a volume and quality amplifier. Dosing strategies of 3–5 g·day^−1^ (with or without a short loading phase) are sufficient to sustain phosphocreatine resynthesis and permit higher training workloads. Morphological adaptations typically emerge after several months of progressive training, consistent with its indirect pathway through enhanced performance rather than direct anabolic signaling [27,28,29,30,63].

Tier 3 covers condition-dependent adjuncts. HMB may be useful during high-load training blocks or energy deficit (~3 g·day^−1^), although expectations should remain moderate given mixed results in well-fed, trained populations [23,24,36,64,65,66]. Omega-3 fatty acids can plausibly sensitize anabolic signaling or aid recovery, particularly where habitual intake of EPA and DHA is low [34,35,36,67,68]. Citrulline and dietary nitrates may improve session quality via enhanced perfusion and tolerance, but morphology-specific evidence remains weak [37,38,69]. Collagen peptides, often paired with vitamin C, appear more relevant to connective-tissue support than direct hypertrophy, yet may indirectly sustain training continuity [34,35,36].

Differentiated Application by Training Level, Sex, and Goal—In untrained or returning individuals, foundational supplementation (adequate protein/EAA and, where appropriate, creatine) supports early anabolic sensitivity and accelerates morphological adaptation [9,10,11,12,13,14,27,28,29,30]. In well-trained adults, the focus should shift toward optimizing per-meal leucine distribution, maintaining creatine saturation, and employing adjuncts such as omega-3s or collagen primarily to sustain training quality and connective-tissue resilience [34,35,36,67]. Sex differences appear minor for morphology outcomes but may influence recovery kinetics and tolerance to high training volumes [19,20]. For athletes aiming at muscle gain, maximizing daily protein distribution and phosphocreatine availability remains central, whereas maintenance or recomposition phases may prioritize recovery facilitation through collagen and omega-3 co-supplementation. Such differentiation highlights that supplement efficacy is context- and goal-dependent rather than universal.

Finally, quality and safety considerations apply across all tiers. Third-party testing, label accuracy, and individual tolerability remain essential, while supplementation strategies should always be integrated with total diet and periodized training [9,10,11,12,13,14,27,28,29,30]. Conceptualized in this way, supplementation does not replace fundamental training principles but can support or amplify them under the right nutritional and physiological conditions.

While the tiered model offers a practical heuristic for interpreting supplement classes, it remains an interpretive construct pending empirical validation. Future research employing standardized imaging, factorial supplementation designs, and direct workload–morphology linkage is needed to test and refine the hierarchy proposed here.

### 4.3. Safety and Ethical Considerations

Beyond efficacy, the safety profile of nutritional supplements in athletic practice warrants equal attention. Reports have documented undeclared substances, mislabeled dosages, or contaminants in commercially available products, raising potential health risks and concerns regarding anti-doping compliance. While the present synthesis emphasized efficacy as captured by morphology-direct outcomes, the scarcity of long-term safety data—particularly in elite athletes—limits firm conclusions on chronic use. For this reason, independent replication, transparent labeling, and third-party testing are critical safeguards. Ethical considerations also extend to ensuring that supplements are positioned as adjuncts to, rather than replacements for, progressive training and balanced nutrition. Integrating efficacy with safety, regulatory oversight, and fair-play principles is essential to translate research findings into responsible sport practice.

### 4.4. Limitations, Measurement Issues, and Research Gaps

This present synthesis is narrative in design, without protocol registration or formal meta-analysis, and thus cannot provide pooled effect sizes or quantitative bias diagnostics. The inclusion of systematic search and screening procedures aimed to improve transparency rather than to imply formal systematic status. Consequently, the absence of preregistration and quantitative synthesis should be interpreted within the context of a narrative evidence integration, where mechanistic coherence rather than statistical pooling guides inference. The emphasis has been on mechanistic interpretation and morphology-direct outcomes, but the absence of systematic synthesis inevitably limits precision. While this approach is appropriate for an integrative framework, it also leaves open the possibility of selection bias and underlines the need for preregistered systematic reviews in the future.

Measurement heterogeneity represents a second major limitation. Ultrasound was the most frequently applied technique, yet often restricted to single anatomical sites with modest reliability reporting, which constrains generalizability. MRI-based trials, although methodologically stronger, remain relatively scarce and unevenly distributed across supplement classes. Inter-operator variability, landmarking differences, and inconsistent reporting of error margins collectively reduce the comparability of trials that otherwise appear similar on the surface. Without harmonized imaging protocols, conclusions about morphological change remain qualified.

Program heterogeneity further complicates interpretation. Training interventions varied widely in volume, progression, supervision, and duration, making it difficult to isolate supplementation effects. Short blocks are particularly problematic, as they may capture early compositional shifts without allowing morphological divergence to manifest. This limits interpretability across creatine and HMB trials, where program quality directly mediates the plausibility of morphological outcomes.

Moderator variables add yet another layer of complexity. Apparent inconsistencies across the literature often reflect differences in sex distribution, training background, or energy balance rather than genuine contradictions in supplement efficacy. Protein and EAA effects clustered in untrained or undernourished cohorts, whereas trained adults with optimized intake frequently exhibited null outcomes. Creatine-related gains appeared most consistent in young male participants under progressive overload, but were less pronounced in mixed-sex or older cohorts. HMB showed context-dependent utility under caloric deficit or deliberate overreaching, but largely neutral effects in eucaloric, resistance-trained settings. Adjuncts such as omega-3 fatty acids occasionally produced small morphological signals in older or nutritionally compromised populations yet were negligible in healthy young adults. Future trials should therefore predefine and report subgroup analyses by these moderators to clarify under which conditions supplementation meaningfully alters hypertrophy trajectories.

These limitations point to clear research gaps. Priority should be given to factorial or “stack” trials with morphology endpoints, standardized imaging protocols and reliability reporting, and larger, adequately powered cohorts. Longitudinal designs linking performance-mediated mechanisms (e.g., creatine-driven workload) to direct morphological outcomes are also needed. Addressing these gaps will require stronger methodological rigor, integration of baseline dietary assessment, and preregistration of analytic plans to reduce selective reporting and strengthen the evidence base.

### 4.5. Standardizing Morphological Assessments (Recommendations)

The future strength of evidence on supplementation and hypertrophy will depend less on the number of trials conducted than on the rigor with which outcomes are assessed and reported. Standardization of morphological assessments is therefore essential. To operationalize this recommendation, a minimum reporting framework is proposed in Table 6.

Adherence to such standards would minimize operator-dependent variability, sharpen the distinction between true hypertrophy and methodological noise, and facilitate cross-study synthesis. Exact imaging sites should be specified, alongside reliability metrics and inter-operator error, to permit replication and comparability across studies. Pre-registration of measurement plans would further reduce selective reporting and improve transparency. In parallel, reporting of resistance-training programs must include set-level details—load, volume, proximity to failure, and progression—so that morphological outcomes can be correctly attributed to the interaction between training and supplementation. Finally, both morphology-direct and performance endpoints should be presented together, allowing mediation analyses that clarify whether supplements act primarily through enhanced workload, anti-catabolic effects, or direct anabolic signaling.

To enable such mediation, a parallel framework is needed for documenting performance variables with the same rigor applied to imaging. Table 7 outlines minimum reporting elements that allow performance outcomes to be paired with morphological endpoints in a reproducible manner.

Together, these dual frameworks—Table 6 for imaging and Table 7 for performance variables—provide the methodological scaffolding needed to resolve the current ambiguity in supplementation trials. By aligning the precision of morphological assessments with equally rigorous documentation of training and performance, future studies will be able to disentangle whether supplements exert their influence through improved workload, reduced catabolism, or direct anabolic signaling. Taken together, such standardization will not only strengthen internal validity but also enable meaningful synthesis across trials, thereby elevating the overall quality of evidence on supplementation and hypertrophy.

## 5. Conclusions

In resistance-trained adults, protein and essential amino acids form the nutritional foundation for hypertrophy-oriented supplementation, with benefits most evident when baseline intake or per-meal leucine exposure is insufficient. Creatine monohydrate contributes indirectly by sustaining phosphocreatine availability and enabling higher training volume and quality, with morphological effects emerging in sufficiently long and progressive programs. HMB shows conditional utility under high training stress or energy deficit, while adjuncts such as omega-3, citrulline/nitrates, and collagen function primarily as facilitators of training tolerance, recovery, or connective-tissue adaptation rather than as direct drivers of hypertrophy. Across supplement classes, ultrasound and MRI outcomes provide greater specificity for morphological adaptation than body-composition surrogates, underscoring the importance of standardized imaging protocols in future trials.

Collectively, the evidence supports a tiered model of supplementation: protein/EAA as the foundation, creatine as a volume amplifier, HMB as a conditional anti-catabolic agent, and adjuncts as provisional facilitators. Implementation should always be integrated with progressive resistance training and adequate total dietary intake. Future work should prioritize factorial designs, standardized imaging, and adequately powered cohorts to clarify interactions among supplements, training, and morphology outcomes.

## Figures and Tables

**Table 1:** Representative trials with direct imaging of hypertrophy (ultrasound/MRI/CT) during resistance training plus nutritional supplementation in adults.

Supplement & Comparator	Population (N/Sex/Age/Training Status)	RT Program & Duration	Imaging (Site)	Dose & Timing	ΔMT/CSA (Primary Outcome)
**Creatine vs. placebo** (ID 14)	30 M; 22 ± 2 y; Trained	Split 5 d/ wk supervised; 6 wks	US multisite (upper/lower)	~0.1 g·kg^−1^·d^−1^ intra-workout	↑ MT over time in both; no between-group difference; strength ↑ with creatine
**Creatine vs. caffeine vs. combo vs. placebo** (ID 16)	32 M/F; 24 ± 3 y; Trained	Total-body 4 d/wk; 6 wks	US (knee extensors; elbow flexors/extensors)	~0.1 g·kg^−1^ pre-workout; caffeine 3 mg·kg^−1^	↑ MT knee extensors with creatine; others ≈ NS
**HMB-FA vs. placebo** (ID 23)	40 M; 23 ± 2 y; Trained	Periodized RT with overreaching; 12 wks	US (quadriceps MT)	3 g·d^−1^ (split doses)	↑ MT and ↑ LBM vs. placebo; strength/power ↑
**Whey/Soy/Leucine vs. Placebo** (ID 2)	28 M; 21 ± 3 y; Untrained	Supervised RT; 12 wks	US (VL thickness)	2×/day; ~3 g leucine/serving	Time effect on VL; no supplement effect; satellite cells ↑ with whey
**Whey vs. placebo** (ID 11)	26 M/F; 25 ± 4 y; Mixed	≥3 mo RT; supervised; 12 wks	US (biceps; VL/VI/RF)	Post-exercise; daily dose	Local VI ↑ with whey; otherwise NS
**Pea protein vs. Whey vs. placebo** (ID 5)	24 M; 20 ± 2 y; Untrained	Upper-body focus; 12 wks	US (biceps MT)	25 g ×2/day (incl. post-session)	↑ biceps all groups; Pea > placebo in weakest subgroup; Pea ≈ Whey
**Hydrolyzed whey vs.** **CHO** (ID 9)	18 M; 24 ± 3 y; Trained	Concentric vs. eccentric emphasis; 12 wks	MRI (quadriceps CSA; tendon)	~0.3 g·kg^−1^ post-session	Whey H > CHO for muscle & tendon hypertrophy
**Greek yogurt vs. CHO** (ID 6)	20 M; 19 ± 2 y; Untrained	Supervised RT; 12 wks	US (biceps MT)	~20 g protein/serving peri-workout & snacks	↑ biceps MT > CHO; strength ↑
**Whey vs. collagen (leucine-equated)** (ID 12)	22 M/F; 21 ± 2 y; Untrained	Supervised RT; 10 wks	US (arm/thigh MT)	Iso-leucine doses	Whey > collagen for ↑ MT during RT

**Notes:** Each row in this table corresponds to a single randomized or controlled trial extracted directly from the literature summarized in Supplementary Table S1. Dosing, timing, and outcome values (ΔMT/CSA) are reproduced verbatim from the original trial reports, not averaged or re-computed. In-table data are representative rather than aggregated, selected to illustrate the range of morphological outcomes across supplement categories. Complete reference details for all studies appear in Supplementary Table S1 (IDs 1–46). US = ultrasound. ID numbers in parentheses correspond to the specific randomized or controlled trials listed in Supplementary Table S1; MRI = magnetic resonance imaging; MT = muscle thickness; CSA = cross-sectional area. ↑ indicates an increase relative to baseline or control.

**Table 2:** Effect-centered outcomes from randomized or controlled trials with direct imaging (US/MRI) endpoints.

Study	Imaging (Site[s])	Δ Treat (Summary)	Δ Control (Summary)	Between-Group Outcome (ΔΔ)	Effect Size (Approx.)	Reliability (ICC/TE)	Assessor Blinded	Overall RoB2
**Creatine vs. placebo**	US multisite (upper/lower)	↑ MT both groups	↑ MT both groups	n.s.	small, n.s.	NR	Y	Some concerns
**Creatine vs. caffeine vs. placebo**	US knee extensors + EF	↑ MT (knee extensors)	≈NS	small ΔΔ	small	ICC NR	NR	Some concerns
**HMB-FA vs. placebo**	US quadriceps	↑ MT, ↑ LBM, ↑ strength	slight ↑MT	significant ΔΔ (*p* < 0.05)	medium	ICC NR	NR	High
**Whey/Soy/** **Leucine vs. placebo**	US VL	↑ VL	↑ VL	n.s.	small, n.s.	Single-site US	Y	Some concerns
**Whey vs. placebo**	US biceps + VL/VI/RF	↑ VI thickness	≈NS	small ΔΔ (VI)	small	Variable reliability	NR	Some concerns
**Pea vs. whey vs. placebo**	US biceps	↑ biceps MT; Pea > placebo	↑ biceps MT	Pea > placebo; Pea ≈ whey	small	Reported consistent	Y	Low
**Hydrolyzed whey vs. CHO**	MRI quadriceps CSA + tendon	↑ CSA, tendon hypertrophy	smaller ↑	Whey > CHO	medium	Standardized MRI	Y	Low
**Greek yogurt vs. CHO**	US biceps	↑ biceps MT; ↑ strength	↑ biceps MT (less)	Yogurt > CHO	small–medium	NR	NR	Some concerns
**Whey vs. collagen**	US arm + thigh	↑ MT (whey)	smaller ↑ MT	Whey > collagen	small–medium	ICC NR	NR	Some concerns

**Notes:** Δ = within-group change; ΔΔ = between-group difference-in-change; MT = muscle thickness; CSA = cross-sectional area; n.s. = not significant; ICC = intraclass correlation coefficient; TE = Typical Error; RoB2 = Risk of Bias (Low/Some concerns/High); ↑ indicates an increase relative to baseline or control.

**Table 3:** Mechanisms, typical dosing/timing, morphology-specific evidence, and consistency by supplement.

Supplement	Primary Mechanistic Node(s)	Typical Dosing (Range)	Typical Timing	Morphology (US/MRI) Evidence in Trained Adults	Consistency	Key Caveats
**Protein/EAA (Leucine)**	MPS/mTORC1; translational efficiency; leucine threshold	~0.3 g·kg^−1^· meal^−1^ or 2–3 g leucine/meal; 3–5 meals/day	Pre/ Post RT; distributed	Positive when baseline intake/distribution is inadequate; diminishing returns when already optimized	Moderate to High	Endpoint choice; per-meal distribution; baseline intake; ceiling effect at high protein
**Creatine Monohydrate**	PCr buffering → set quality & training volume	3–5 g/day (±short loading ~0.3 g·kg^−1^·day^−1^ × 5–7 days)	Daily; align with RT blocks	Small–moderate gains with long, progressive RT; neutral when blocks are short or under-progressed	Moderate	Program duration; early water shifts; inter-individual variability; imaging site
**HMB** **(FA or Ca-salt)**	Anti-catabolic (proteolysis/ autophagy); membrane stability	~3 g /day (FA or Ca-salt)	Daily; consider high-stress or deficit	Context-dependent; mixed/neutral in eucaloric, highly trained settings	Low to Moderate	Context-dependent; formulation differences; energy status; industry sponsorship
**Omega-3 Fatty Acids**	Membrane/ inflammation; anabolic sensitivity	~1–3 g /day EPA + DHA	Daily	Limited/heterogeneous direct morphology outcomes; may facilitate training/recovery	Low	Dependent on adequate protein and overload; small samples; heterogeneous dosing
**Citrulline/** **Nitrates**	NO pathway; perfusion/ tolerance	6–8 g L-citrulline; dietary nitrates	Pre - exercise	Inconsistent morphology; performance effects more common	Low	Large inter-study variation; acute vs. chronic effects; weak morphology linkage
**Collagen (+Vitamin C)**	Connective-tissue support	10–15 g /day	Daily; ± pre-rehab/RT	Limited direct CSA gains; indirect support to training continuity	Low	Outcome alignment; Vit C timing; often inferior to whey comparators; targeted use-cases
**Protein/EAA (Leucine)**	MPS/mTORC1; translational efficiency; leucine threshold	~0.3 g·kg^−1^· meal^−1^ or 2–3 g leucine/meal; 3–5 meals/day	Pre/ Post RT; distributed	Positive when baseline intake/distribution is inadequate; diminishing returns when already optimized	Moderate to High	Endpoint choice; per-meal distribution; baseline intake; ceiling effect at high protein
**Creatine Monohydrate**	PCr buffering → set quality & training volume	3–5 g/day (±short loading ~0.3 g·kg^−1^·day^−1^ × 5–7 days)	Daily; align with RT blocks	Small–moderate gains with long, progressive RT; neutral when blocks are short or under-progressed	Moderate	Program duration; early water shifts; inter-individual variability; imaging site
**HMB** **(FA or Ca-salt)**	Anti-catabolic (proteolysis/ autophagy); membrane stability	~3 g /day (FA or Ca-salt)	Daily; consider high-stress or deficit	Context-dependent; mixed/neutral in eucaloric, highly trained settings	Low to Moderate	Context-dependent; formulation differences; energy status; industry sponsorship
**Omega-3 Fatty Acids**	Membrane/ inflammation; anabolic sensitivity	~1–3 g /day EPA + DHA	Daily	Limited/heterogeneous direct morphology outcomes; may facilitate training/recovery	Low	Dependent on adequate protein and overload; small samples; heterogeneous dosing
**Citrulline/** **Nitrates**	NO pathway; perfusion/ tolerance	6–8 g L-citrulline; dietary nitrates	Pre - exercise	Inconsistent morphology; performance effects more common	Low	Large inter-study variation; acute vs. chronic effects; weak morphology linkage
**Collagen (+Vitamin C)**	Connective-tissue support	10–15 g /day	Daily; ± pre-rehab/RT	Limited direct CSA gains; indirect support to training continuity	Low	Outcome alignment; Vit C timing; often inferior to whey comparators; targeted use-cases

**Notes:** Consistency ratings reflect qualitative synthesis across randomized or controlled trials with ultrasound/MRI endpoints. Abbreviations: US = ultrasound; MRI = magnetic resonance imaging; CSA = cross-sectional area; EAA = essential amino acids; PCr = phosphocreatine.

**Table 4:** Quantitative synthesis of supplement classes with imaging endpoints.

Supplement Class	Imaging RCTs (*n*)	Median Duration (wk)	Typical ΔΔ (US/MRI)	Typical Δ% Hypertrophy	Signal Strength
**Protein/EAA (Leucine)**	~9	10–12	+1–2 mm MT (vastus lateralis, biceps)	+3–5% vs. controls (when baseline intake <1.6 g·kg^−1^·day^−1^)	Strong, but conditional
**Creatine monohydrate**	~7	8–12	+1.5–2.0 mm MT or +0.3–0.5 cm^2^ CSA (KE, EF)	+5–7% hypertrophy over controls in long programs	Moderate-to-strong
**HMB (FA/Ca-salt)**	~3	12	FA trial: +2–3 mm quadriceps MT; others neutral	+4–6% in stressed/deficit states; ≈0% in eucaloric trained	Weak-to-moderate, context-dependent
**Omega-3 fatty acids**	2	8–12	≈n.s. for MT/CSA in young trained adults	≤1% Δ vs. controls	Weak
**Citrulline/Nitrates**	1–2	6–8	No reproducible MT/CSA effect	0%	Very weak
**Collagen (+Vit C)**	2	8–10	Neutral for MT; ↑ tendon CSA ~5–10%	0% muscle; +5–10% tendon	Weak (connective tissue support)

**Notes:** ΔΔ = between-group difference-in-change; Δ% = relative hypertrophy effect vs. control; MT = muscle thickness; CSA = cross-sectional area; KE = knee extensors; EF = elbow flexors; FA = free acid formulation of HMB; n.s. = not significant; ↑ indicates an increase relative to baseline or control.

**Table 5:** Risk-of-Bias (RoB 2) appraisal for trials with ultrasound/MRI endpoints.

Trial	Randomization	Outcome Measurement	Reporting Bias	Notes (Sponsorship/Blinding)	Overall Risk
**Creatine vs. placebo**	Low	Some concerns (single-site US)	Low	Independent; assessor blinded	Some concerns
**Creatine vs. caffeine vs. combo vs. placebo**	Some concerns	Some concerns (limited sites; ICC NR)	Low	Independent; blinding NR	Some concerns
**HMB-FA vs. placebo**	Some concerns	Some concerns (industry involvement; ICC NR)	High	Industry-sponsored; assessor NR	High
**Whey/Soy/Leucine vs. placebo**	Low	Some concerns (single-muscle US)	Low	Independent; assessor blinded	Some concerns
**Whey vs. placebo**	Low	Some concerns (multi-site US; variable reliability)	Low	Independent; blinding NR	Some concerns
**Pea protein vs. Whey vs. placebo**	Low	Low (US; biceps thickness; consistent reporting)	Low	Independent	Low
**Hydrolyzed whey vs. CHO**	Low	Low (MRI standardized, tendon + muscle CSA)	Low	Independent; assessor blinded	Low
**Greek yogurt vs. CHO**	Low	Some concerns (single-site US)	Low	Independent; blinding NR	Some concerns
**Whey vs. collagen (leucine-equated)**	Low	Some concerns (US; variable sites; ICC NR)	Low	Independent; blinding NR	Some concerns

**Notes:** ICC = intraclass correlation coefficient; NR = not reported. Judgments based on Cochrane RoB 2 domains (randomization, deviations from interventions, missing data, measurement of outcome, reporting). Sponsorship and blinding were extracted from trial reports where disclosed.

**Table 6:** Minimum reporting standards for morphology-direct imaging in hypertrophy trials.

Domain	Minimum Reporting Requirement
**Anatomical site**	Exact landmark (e.g., vastus lateralis at 50% femur length); specify side and limb dominance.
**Probe handling (US)**	Angle of insonation, applied pressure, coupling medium (gel, stand-off pad).
**Timing/participant state**	Standardize time of day, hydration, prior exercise, and nutrition before scans.
**Repetition/averaging**	Acquire ≥3 images per site recommended; report procedure for averaging values.
**Reliability metrics**	Provide intra- and inter-rater ICC; Typical Error (TE) or coefficient of variation (CV).
**Assessor blinding**	State explicitly whether imaging assessor was blinded to allocation and time point.
**Data reporting**	Report baseline and post values (mean ± SD), within-group Δ, between-group ΔΔ with 95% CI.
**Units & transparency**	Use standardized units (mm for MT; cm^2^ for CSA); pre-register measurement protocol; report device make/model.

**Notes:** The items listed represent minimum reporting elements that improve comparability and reproducibility of morphology-direct imaging outcomes. Abbreviations: US = ultrasound; MT = muscle thickness; CSA = cross-sectional area; ICC = intraclass correlation coefficient; TE = typical error.

**Table 7:** Minimum reporting standards for performance–morphology integration.

Domain	Reporting Requirement
**Training load & volume**	Total sets, reps, and load lifted per week (absolute and relative to baseline).
**Intensity & effort**	%1RM or RIR (repetitions in reserve) for each session.
**Session density**	Rest intervals and total session duration.
**Progression**	Week-to-week changes in load, volume, or intensity.
**Performance outcomes**	Strength (1RM, isometric MVC), endurance, power (jump, sprint).
**Morphological outcomes**	US/MRI: MT, CSA with exact site and reliability indices.
**Linking variables**	Paired reporting of workload and morphology to permit mediation analyses.

**Notes:** RIR = repetitions in reserve; 1RM = one-repetition maximum; MVC = maximal voluntary contraction; MT = muscle thickness; CSA = cross-sectional area.

## Data Availability

No new data were generated or analyzed in this study. Data sharing is not applicable to this article.

## Supplementary Materials

The following supporting information can be downloaded at: https://www.mdpi.com/article/10.3390/nu17223603/s1, Table S1. Randomized controlled trials investigating nutritional supplementation and muscle hypertrophy assessed with imaging methods in adults. References [71,72,73,74,75,76,77,78,79,80,81,82,83,84,85,86,87,88,89,90,91,92,93,94,95,96,97,98,99,100,101,102,103,104,105,106,107,108,109,110,111,112,113] were cited in Supplementary Materials.

## References

[1] Iraki J., Fitschen P., Espinar S., Helms E. (2019). Nutrition Recommendations for Bodybuilders in the Off-Season. Sports.

[2] Mazzilli M., Macaluso F., Zambelli S., Picerno P., Iuliano E. (2021). The Use of Dietary Supplements in Fitness Practitioners: A Cross-Sectional Observation Study. Int. J. Environ. Res. Public Health.

[3] Knapik J.J., Steelman R.A., Hoedebecke S.S., Austin K.G., Farina E.K., Lieberman H.R. (2016). Prevalence of Dietary Supplement Use by Athletes: Systematic Review and Meta-Analysis. Sports Med..

[4] Tidmas V., Turner A., Koropsak D., Tinsley G.M. (2022). Nutritional and Non-Nutritional Strategies in Bodybuilding. Int. J. Environ. Res. Public Health.

[5] Cruz-Jentoft A.J., Bahat G., Bauer J., Boirie Y., Bruyère O., Cederholm T., Cooper C., Landi F., Rolland Y., Sayer A.A. (2019). Sarcopenia: Revised European Consensus on Definition and Diagnosis (EWGSOP2). Age Ageing.

[6] Balakrishnan R., Thurmond D.C. (2022). Mechanisms by Which Skeletal Muscle Myokines Regulate Metabolism. Int. J. Mol. Sci..

[7] Manescu D.C. (2016). Nutritional tips for muscular mass hypertrophy. Marathon.

[8] McKay M.J., Weber K.A., Wesselink E.O., Smith Z.A., Abbott R., Anderson D.B., Ashton-James C.E., Atyeo J., Beach A.J., Burns J. (2024). MuscleMap: An Open-Source, Community-Supported Consortium for Whole-Body Quantitative MRI of Muscle. J. Imaging.

[9] Burke R., Piñero A., Coleman M., Mohan A., Sapuppo M., Augustin F., Aragon A.A., Candow D.G., Forbes S.C., Swinton P. (2023). The Effects of Creatine Supplementation Combined with Resistance Training on Regional Measures of Muscle Hypertrophy: A Systematic Review with Meta-Analysis. Nutrients.

[10] Gonzalez A.M., Townsend J.R., Pinzone A.G., Hoffman J.R. (2023). Supplementation with Nitric Oxide Precursors for Strength Performance: A Review of the Current Literature. Nutrients.

[11] Hooijmans M.T., Schlaffke L., Bolsterlee B., Schlaeger S., Marty B., Mazzoli V. (2024). Compositional and Functional MRI of Skeletal Muscle: A Review. J. Magn. Reson. Imaging.

[12] Sinha U., Sinha S. (2024). Magnetic Resonance Imaging Biomarkers of Muscle. Tomography.

[13] Manescu D.C. (2010). Alimentaţia în Fitness şi Bodybuilding.

[14] Hirabara S.M., Marzuca-Nassr G.N., Cury-Boaventura M.F. (2024). Nutrition and Exercise Interventions on Skeletal Muscle Physiology, Injury and Recovery: From Mechanisms to Therapy. Nutrients.

[15] Manescu D.C. (2025). Nutriție Ergogenă, Suplimentație și Performanță.

[16] Kuikman M.A., Smith E., McKay A.K.A., McCormick R., Ackerman K.E., Harris R., Elliott-Sale K.J., Stellingwerff T., Burke L.M. (2025). Impact of Acute Dietary and Exercise Manipulation on Next-Day RMR Measurements and DXA Body Composition Estimates. Med. Sci. Sports Exerc..

[17] Kojima C., Namma-Motonaga K., Kamei A., Takahashi Y., Ishibashi A., Takahashi H. (2025). Dynamics of Muscle Glycogen Increase in Brachial and Thigh Muscles with Carbohydrate Loading. Eur. J. Appl. Physiol..

[18] Tavoian D., Ampomah K., Amano S., Law T.D., Clark B.C. (2019). Changes in DXA-Derived Lean Mass and MRI-Derived Cross-Sectional Area of the Thigh Are Modestly Associated. Sci. Rep..

[19] Stokes T., Tyler C., Goosey-Tolfrey V., Burniston J., English C., Phillips B.E., Wilkinson D.J., Atherton P.J. (2021). Methodological Considerations for and Validation of the Use of B-Mode Ultrasonography to Estimate Changes in Muscle Size. Physiol. Rep..

[20] Ruple B.A., Smith M.A., Osburn S.C., Sexton C.L., Godwin J.S., Edison J.L., Poole C.N., Stock M.S., Fruge A.D., Young K.C. (2022). Comparisons between skeletal muscle imaging techniques and histology in tracking midthigh hypertrophic adaptations following 10 weeks of resistance training. J. Appl. Physiol..

[21] Engelke K., Chaudry O., Gast L., Ab Eldib M., Wang L., Laredo J.-D., Nagel A.M. (2023). Magnetic Resonance Imaging Techniques for the Quantitative Analysis of Skeletal Muscle: State of the Art. J. Orthop. Translat..

[22] Alic L., Griffin J.F., Eresen A., Kornegay J.N., Ji J.X. (2021). Using MRI to quantify skeletal muscle pathology in Duchenne muscular dystrophy: A systematic mapping review. Muscle Nerve.

[23] Zamosteanu D., Filip N., Trandafir L.M., Ţarcă E., Pertea M., Bordeianu G., Bernic J., Heredea A.M., Cojocaru E. (2025). Current Data on the Role of Amino Acids in the Management of Obesity in Children and Adolescents. Int. J. Mol. Sci..

[24] Jeong D., Park K., Lee J., Choi J., Du H., Jeong H., Li L., Sakai K., Kang S. (2024). Effects of Resistance Exercise and Essential Amino Acid Intake on Muscle Quality, Myokine, and Inflammation Factors in Young Adult Males. Nutrients.

[25] Churchward-Venne T.A., Breen L., Di Donato D.M., Hector A.J., Mitchell C.J., Moore D.R., Stellingwerff T., Breuille D., Offord E.A., Baker S.K. (2014). Leucine Supplementation of a Low-Protein Meal Enhances Myofibrillar Protein Synthesis in Young Men: A Randomized Crossover Trial. Am. J. Clin. Nutr..

[26] Jäger R., Kerksick C.M., Campbell B.I., Cribb P.J., Wells S.D., Skwiat T.M., Purpura M., Ziegenfuss T.N., Ferrando A.A., Arent S.M. (2017). International Society of Sports Nutrition Position Stand: Protein and Exercise. J. Int. Soc. Sports Nutr..

[27] Branch J.D. (2003). Effect of creatine supplementation on body composition and performance: A meta-analysis. Int. J. Sport Nutr. Exerc. Metab..

[28] Candow D.G., Chilibeck P.D., Burke D.G., Mueller K.D., Lewis J.D. (2011). Effect of Different Frequencies of Creatine Supplementation on Muscle Size and Strength in Young Adults. J. Strength Cond. Res..

[29] Dinan N.E., Forbes S.C., Stout J.R., Cooke M., Taylor K.-L., Hannan A., Pulido E., Wilborn C., Ergogenic Adaptations Research Group (2022). Effects of creatine monohydrate timing on resistance training adaptations and body composition. Front. Sports Act. Living.

[30] Roschel H., Gualano B., Ostojic S.M., Rawson E.S. (2021). Creatine Supplementation and Brain Health. Nutrients.

[31] Rathmacher J.A., Pitchford L.M., Stout J.R., Townsend J.R., Jäger R., Kreider R.B., Campbell B.I., Kerksick C.M., Harty P.S., Candow D.G. (2025). International Society of Sports Nutrition Position Stand: β-Hydroxy-β-Methylbutyrate (HMB). J. Int. Soc. Sports Nutr..

[32] Bideshki M.V., Behzadi M., Jamali M., Jamilian P., Zarezadeh M., Pourghassem Gargari B. (2025). Ergogenic Benefits of β-Hydroxy-β-Methyl Butyrate (HMB) Supplementation on Body Composition and Muscle Strength: An Umbrella Review of Meta-Analyses. J. Cachexia Sarcopenia Muscle.

[33] Courel-Ibáñez J., Vetrovsky T., Dadova K., Pallarés J.G., Steffl M. (2019). Health Benefits of β-Hydroxy-β-Methylbutyrate (HMB) Supplementation in Addition to Physical Exercise in Older Adults: A Systematic Review with Meta-Analysis. Nutrients.

[34] Brown K., Persinger A., Pryke A., Lin J., Wallace N., Chizhikov D., Puppa M. (2025). Effects of omega-3- and omega-6-rich high-fat diets on skeletal muscle protein degradation signaling in glucocorticoid-treated mice. Int. J. Funct. Nutr..

[35] Son W., Brown K., Persinger A., Pryke A., Lin J., Powell Z., Wallace N., van der Merwe M., Puppa M. (2023). Effect of Omega-3 Rich High-Fat Diet on Markers of Tissue Lipid Metabolism in Glucocorticoid-Treated Mice. Int. J. Mol. Sci..

[36] Da Boit M., Sibson R., Sivasubramaniam S., Meakin J.R., Greig C.A., Aspden R.M., Thies F., Jeromson S., Hamilton D.L., Speakman J.R. (2017). Sex-Specific Effects of *n*-3 PUFA on Muscle Function and Quality in Older Adults: A Randomized Controlled Trial. Am. J. Clin. Nutr..

[37] Alsharif N.S., Clifford T., Alhebshi A., Rowland S.N., Bailey S.J. (2023). Effects of Dietary Nitrate Supplementation on Performance during Single and Repeated Bouts of Short-Duration High-Intensity Exercise: A Systematic Review and Meta-Analysis of Randomised Controlled Trials. Antioxidants.

[38] Martínez-Puig D., Chevalier X., Manfredini D., Ornetti P., Henrotin Y. (2023). Collagen Supplementation for Joint Health: Mechanisms and Clinical Evidence. Nutrients.

[39] Hudson J.L., Bergia R.E., Campbell W.W. (2020). Protein Distribution and Muscle-Related Outcomes: Does the Evidence Support the Concept?. Nutrients.

[40] Layman D.K. (2024). Impacts of Protein Quantity and Distribution on Body Composition. Front. Nutr..

[41] Goldman D.M., Warbeck C.B., Karlsen M.C. (2024). Completely Plant-Based Diets That Meet Energy Requirements for Resistance Training Can Supply Enough Protein and Leucine to Maximize Hypertrophy and Strength in Male Bodybuilders: A Modeling Study. Nutrients.

[42] Smith G.I., Atherton P., Reeds D.N., Mohassel P., Rankin D., Mitchell W.K., Kumar V., Narayan D.S., Mittendorfer B. (2011). Omega-3 Fatty Acids Increase the Rate of Muscle Protein Synthesis in Older Adults: A Randomized Controlled Trial. Am. J. Clin. Nutr..

[43] Amiri E., Sheikholeslami-Vatani D. (2023). The role of resistance training and creatine supplementation on oxidative stress, antioxidant defense, muscle strength, and quality of life in older adults. Front. Public Health.

[44] Therdyothin A., Prokopidis K., Galli F., Witard O.C., Isanejad M. (2025). The effects of omega-3 polyunsaturated fatty acids on muscle and whole-body protein synthesis: A systematic review and meta-analysis. Nutr. Rev..

[45] Sousa-Silva R., Cholewa J.M., de Araújo Pessôa K., Xia Z., Lauver J.D., Rossi F.E., Zanchi N.E. (2023). Creatine supplementation combined with blood flow restriction training enhances muscle thickness and performance: A randomized, placebo-controlled, and double-blind study. Appl. Physiol. Nutr. Metab..

[46] Wilson J.M., Lowery R.P., Joy J.M., Andersen J.C., Wilson S.M.C., Stout J.R., Duncan N., Fuller J.C., Baier S.M., Naimo M.A. (2014). The Effects of 12 Weeks of Beta-Hydroxy-Beta-Methylbutyrate Free Acid Supplementation on Muscle Mass, Strength, and Power in Resistance-Trained Individuals: A Randomized, Double-Blind, Placebo-Controlled Study. Eur. J. Appl. Physiol..

[47] Jakubowski J.S., Wong E.P.T., Nunes E.A., Noguchi K.S., Vandeweerd J.K., Murphy K.T., Morton R.W., McGlory C., Phillips S.M. (2019). Equivalent Hypertrophy and Strength Gains in β-Hydroxy-β-Methylbutyrate- or Leucine-Supplemented Men. Med. Sci. Sports Exerc..

[48] Hu Y.-G., Shi J.-H., Yu D.-X., Huang H.-B. (2025). The Effects of β-Hydroxy-β-Methyl Butyrate Supplementation in Surgical Patients: A Systematic Review and Meta-Analysis of Randomized Controlled Trials. Front. Nutr..

[49] Tomczyk M. (2024). Omega-3 Fatty Acids and Muscle Strength—Current State of Knowledge and Future Perspectives. Nutrients.

[50] Shaw G., Lee-Barthel A., Ross M.L., Wang B., Baar K. (2017). Vitamin C-Enriched Gelatin Supplementation before Intermittent Activity Augments Collagen Synthesis. Am. J. Clin. Nutr..

[51] Manescu D.C. (2025). Fitness.

[52] Inacio P.A.Q., Gomes Y.S.M., de Aguiar A.J.N., Lopes-Martins P.S.L., Aimbire F., Leonardo P.S., Sá Filho A.S., Lopes-Martins R.A.B. (2024). The Effects of Collagen Peptides as a Dietary Supplement on Muscle Damage Recovery and Fatigue Responses: An Integrative Review. Nutrients.

[53] Forsting J., Forsting M., Froeling M., Güttsches A.-K., Südkamp N., Roos A., Vorgerd M., Schlaffke L., Rehmann R. (2022). Quantitative muscle MRI captures early fat infiltration in leg muscles: A study on calpainopathy. Sci. Rep..

[54] De Mello R., Ma Y., Ji Y., Du J., Chang E.Y. (2019). Quantitative MRI Musculoskeletal Techniques: An Update. AJR Am. J. Roentgenol..

[55] Chianca V., Vincenzo B., Cuocolo R., Zappia M., Guarino S., Di Pietto F., Del Grande F. (2023). MRI Quantitative Evaluation of Muscle Fatty Infiltration. Magnetochemistry.

[56] Eck B.L., Yang M., Elias J.J., Winalski C.S., Altahawi F., Subhas N., Li X. (2023). Quantitative MRI for Evaluation of Musculoskeletal Disease: Cartilage and Muscle Composition, Joint Inflammation, and Biomechanics in Osteoarthritis. Investig. Radiol..

[57] Emanuelsson E.B., Berry D.B., Reitzner S.M., Arif M., Mardinoglu A., Gustafsson T., Ward S.R., Sundberg C.J., Chapman M.A. (2022). MRI Characterization of Skeletal Muscle Size and Fatty Infiltration in Long-Term Trained and Untrained Individuals. Physiol. Rep..

[58] Ong J.N., Ducker K.J., Furzer B.J., Dymock M., Landers G.J. (2023). Acute exercise affects dual-energy X-ray absorptiometry body composition estimates but not standardised ultrasound measurements of subcutaneous adipose tissue. Clin. Physiol. Funct. Imaging.

[59] Kondo E., Takai E., Sagayama H., Takahashi H. (2023). Comparison of three type(s) of muscle glycogen loading interventions using a very-high-carbohydrate diet in an elite male racewalker: A case report. Phys. Act. Nutr..

[60] Högelin E.R., Thulin K., von Walden F., Fornander L., Michno P., Alkner B. (2022). Reliability and validity of an ultrasound-based protocol for measurement of quadriceps muscle thickness in children. Front. Physiol..

[61] Thanaj M., Basty N., Whitcher B., Sorokin E.P., Liu Y., Srinivasan R., Cule M., Thomas E.L., Bell J.D. (2024). Precision MRI phenotyping of muscle volume and quality at a population scale. Front. Physiol..

[62] Areta J.L., Burke L.M., Ross M.L., Camera D.M., West D.W.D., Broad E.M., Jeacocke N.A., Moore D.R., Stellingwerff T., Phillips S.M. (2013). Timing and distribution of protein ingestion during prolonged recovery from resistance exercise alters myofibrillar protein synthesis. J. Physiol..

[63] Helms E.R., Aragon A.A., Fitschen P.J. (2014). Evidence-Based Recommendations for Natural Bodybuilding Contest Preparation: Nutrition and Supplementation. J. Int. Soc. Sports Nutr..

[64] Kreider R.B., Kalman D.S., Antonio J., Ziegenfuss T.N., Wildman R., Collins R., Candow D.G., Kleiner S.M., Almada A.L., Lopez H.L. (2017). International Society of Sports Nutrition Position Stand: Safety and Efficacy of Creatine Supplementation in Exercise, Sport, and Medicine. J. Int. Soc. Sports Nutr..

[65] Holeček M. (2017). Beta-hydroxy-beta-methylbutyrate supplementation and skeletal muscle in healthy and muscle-wasting conditions. J. Cachexia Sarcopenia Muscle.

[66] Teixeira F.J., Matias C.N., Monteiro C.P., Valamatos M.J., Reis J.F., Batista A., Oliveira A.C., Alves F., Sardinha L.B., Phillips S.M. (2019). No Effect of HMB or α-HICA Supplementation on Training-Induced Changes in Body Composition. Eur. J. Sport Sci..

[67] Deutz N.E.P., Pereira S.L., Hays N.P., Oliver J.S., Edens N.K., Evans C.M., Wolfe R.R. (2013). Effect of β-hydroxy-β-methylbutyrate (HMB) on lean body mass during 10 days of bed rest in older adults. Clin. Nutr..

[68] Lee S.-R., Jo E., Khamoui A.V. (2019). Chronic Fish Oil Consumption with Resistance Training Improves Grip Strength, Physical Function, and Blood Pressure in Community-Dwelling Older Adults. Sports.

[69] Baráth A., Annár D., Györe I., Szmodis M. (2024). The Effects of L-Citrulline and Malic Acid on Substrate Utilisation and Lactate Elimination. Appl. Sci..

[70] Zdzieblik D., Oesser S., Baumstark M.W., Gollhofer A., König D. (2015). Collagen peptide supplementation in combination with resistance training improves body composition and increases muscle strength in elderly sarcopenic men: A randomized controlled trial. Br. J. Nutr..

[71] Reidy P.T., Borack M.S., Markofski M.M., Dickinson J.M., Deer R.R., Husaini S.H., Walker D.K., Igbinigie S., Robertson S.M., Cope M.B. (2016). Protein Supplementation Has Minimal Effects on Muscle Adaptations during Resistance Exercise Training in Young Men: A Double-Blind Randomized Clinical Trial. J. Nutr..

[72] Mobley C.B., Haun C.T., Roberson P.A., Mumford P.W., Kephart W.C., Romero M.A., Osburn S.C., Vann C.G., Young K.C., Beck D.T. (2017). Effects of Whey, Soy or Leucine Supplementation with 12 Weeks of Resistance Training on Strength, Body Composition, and Skeletal Muscle and Adipose Tissue Histological Attributes in College-Aged Males. Nutrients.

[73] Joy J.M., Lowery R.P., Wilson J.M., Purpura M., De Souza E.O., Wilson S.M., Kalman D.S., Dudeck J.E., Jäger R. (2013). The Effects of 8 Weeks of Whey or Rice Protein Supplementation on Body Composition and Exercise Performance. Nutr. J..

[74] Lynch H.M., Wharton C., Johnston C.S., Kris-Etherton P.M., Post R.E., Parkinson A.L., Little R.B., Most M., West S.G., Armamento-Villareal R. (2020). No Significant Differences in Muscle Growth and Strength Development When Consuming Soy and Whey Protein Supplements Matched for Leucine Following a 12-Week Resistance Training Program in Men and Women: A Randomized Trial. Int. J. Environ. Res. Public Health.

[75] Babault N., Païzis C., Deley G., Guérin-Deremaux L., Saniez M.H., Lefranc-Millot C., Allaert F.A. (2015). Pea Proteins Oral Supplementation Promotes Muscle Thickness Gains during Resistance Training: A Double-Blind, Randomized, Placebo-Controlled Clinical Trial vs. Whey Protein. J. Int. Soc. Sports Nutr..

[76] Bridge A., Brown J., Snider H., Nasato M., Prapavessis H. (2019). Greek Yogurt and 12 Weeks of Exercise Training on Strength, Muscle Thickness, and Body Composition in Lean, Untrained, University-Aged Males. Front. Nutr..

[77] Sharp M.H., Lowery R.P., Shields K.A., Lane J.R., Gray J.L., Partl J.M., Hayes D.W., Wilson G.J., Hollmer C.A., Minivich J.R. (2018). The Effects of Beef, Chicken, or Whey Protein after Workout on Body Composition and Muscle Performance. J. Strength Cond. Res..

[78] Naclerio F., Seijo M., Larumbe-Zabala E., Earnest C.P. (2017). Carbohydrates Alone or Mixing with Beef or Whey Protein Promote Similar Training Outcomes in Resistance-Training Males: A Double-Blind, Randomized Controlled Clinical Trial. Int. J. Sport Nutr. Exerc. Metab..

[79] Farup J., Rahbek S.K., Vendelbo M.H., Matzon A., Hindhede J., Bejder A., Ringgaard S., Vissing K. (2014). High-Leucine Whey Protein Hydrolysate Augments Muscle and Tendon Hypertrophy Following 12 Weeks of Resistance Training—Irrespective of Contraction Mode. Scand. J. Med. Sci. Sports.

[80] Vieillevoye S., Poortmans J.R., Duchateau J., Carpentier A. (2010). Effects of a Combined Essential Amino Acids/Carbohydrate Supplementation on Muscle Mass, Architecture and Maximal Strength Following Heavy-Load Training. Eur. J. Appl. Physiol..

[81] Banaszek A., Townsend J.R., Bender D., Vantrease W.C., Marshall A.C., Johnson K.D. (2019). The Effects of Whey vs. Pea Protein on Physical Adaptations Following 8-Weeks of High-Intensity Functional Training (HIFT): A Pilot Study. Sports.

[82] Jacinto J.L., Nunes J.P., Gorissen S.H.M., Capel D.M.G., Bernardes A.G., Ribeiro A.S., Cyrino E.S., Phillips S.M., Aguiar A.F. (2022). Whey Protein Supplementation Is Superior to Leucine-Matched Collagen Peptides to Increase Muscle Thickness During a 10-Week Resistance Training Program in Untrained Young Adults. Int. J. Sport Nutr. Exerc. Metab..

[83] Hartman J.W., Tang J.E., Wilkinson S.B., Tarnopolsky M.A., Lawrence R.L., Fullerton A.V., Phillips S.M. (2007). Consumption of Fat-Free Fluid Milk after Resistance Exercise Promotes Greater Lean Mass Accretion than Does Consumption of Soy or Carbohydrate in Young, Novice, Male Weightlifters. Am. J. Clin. Nutr..

[84] Chilibeck P.D., Stride D., Farthing J.P., Burke D.G. (2004). Effect of Creatine Ingestion after Exercise on Muscle Thickness in Males and Females. Med. Sci. Sports Exerc..

[85] Mills S., Candow D.G., Forbes S.C., Neary J.P., Ormsbee M.J., Antonio J. (2020). Effects of Creatine Supplementation during Resistance Training Sessions in Physically Active Young Adults. Nutrients.

[86] Pakulak A., Muddle T.W.D., Rollo I., Galloway S.D.R., Pritchard H.J., Tallis J. (2022). Effects of Creatine and Caffeine Supplementation during Resistance Training on Body Composition, Strength, Endurance, RPE and Fatigue in Trained Young Adults. J. Diet. Suppl..

[87] Schoenfeld B.J., Aragon A.A., Wilborn C., Urbina S., Hayward S., Krieger J. (2017). Pre- versus Post-Exercise Protein Intake Has Similar Effects on Muscular Adaptations. PeerJ.

[88] Joy J.M., Lowery R.P., Wilson J.M., Purpura M., De Souza E.O., McDonnell E., Wilson S.M.C., Kalman D.S., Dudeck J.E., Jäger R. (2014). Phosphatidic Acid Enhances mTOR Signaling and Resistance Exercise Training Adaptations in Human Skeletal Muscle. Nutr. Metab..

[89] Gonzalez A.M., Sell K.M., Ghigiarelli J.J., Kelly C.F., Shone E.W., Accetta M.R., Baum J.B., Mangine G.T. (2017). Effects of Phosphatidic Acid Supplementation on Muscle Thickness and Strength in Resistance-Trained Men. Appl. Physiol. Nutr. Metab..

[90] Andre T.L., Gann J.J., McKinley-Barnard S.K., Song J.J., Willoughby D.S. (2016). Eight Weeks of Phosphatidic Acid Supplementation in Conjunction with Resistance Training Does Not Differentially Affect Body Composition and Muscle Strength in Resistance-Trained Men. J. Sports Sci. Med..

[91] Escalante G., Alencar M., Haddock B., Harvey P. (2016). The Effects of Phosphatidic Acid Supplementation on Strength, Body Composition, Muscular Endurance, Power, Agility, and Vertical Jump in Resistance Trained Men. J. Int. Soc. Sports Nutr..

[92] Lowery R.P., Joy J.M., Rathmacher J.A., Baier S.M., Fuller J.C., Shelley M.C., Jäger R., Purpura M., Wilson S.M.C., Wilson J.M. (2016). Interaction of Beta-Hydroxy-Beta-Methylbutyrate Free Acid and Adenosine Triphosphate on Muscle Mass, Strength, and Power in Resistance-Trained Individuals. J. Strength Cond. Res..

[93] Townsend J.R., Hart T.L., Haynes J.T., Woods C.A., Toy A.M., Pihera B.C. (2022). Influence of Dietary Nitrate Supplementation on Physical Performance and Body Composition Following Offseason Training in Division I Athletes. J. Diet. Suppl..

[94] Jerger S., Bohm S., Marzilger R., Mersmann F., Arampatzis A. (2022). Effects of Specific Collagen Peptide Supplementation on Tendon and Muscle Adaptations to High-Load Resistance Training: A Randomized Controlled Trial. Scand. J. Med. Sci. Sports.

[95] Balshaw T.G., Funnell M.P., McDermott E., Maden-Wilkinson T.M., Abela S., Quteishat B., Edsey M., James L.J., Folland J.P. (2023). The effect of specific bioactive collagen peptides on function and muscle remodeling during human resistance training. Acta Physiol..

[96] Lee J., Bridge J.E., Clark D.R., Stewart C.E., Erskine R.M. (2023). Collagen Supplementation Augments Changes in Patellar Tendon Properties in Female Soccer Players. Front. Physiol..

[97] Michel J.M., Lievense K.K., Norton S.C., Costa J.V., Alphin K.H., Bailey L.A., Miller G.D. (2022). The Effects of Graded Protein Intake in Conjunction with Resistance Training on Muscle Mass, Strength, and Physical Function in Older Adults: A Randomized Trial. Nutrients.

[98] Fraschetti E.C., Abdul-Sater A.A., Perry C.G.R., Josse A.R. (2025). R. Resistance Exercise Training and Greek Yogurt Consumption Modulate Markers of Systemic Inflammation in Healthy Young Males—A Secondary Analysis of a Randomized Controlled Trial. Nutrients.

[99] Joy J.M., Vogel R.M., Shane Broughton K., Kudla U., Kerr N.Y., Davison J.M. (2018). Daytime and Nighttime Casein Supplements Similarly Increase Muscle Size and Strength in Response to Resistance Training. J. Int. Soc. Sports Nutr..

[100] Babault N., Deley G., Le Ruyet P., Morgan F., Allaert F.A. (2014). Effects of Soluble Milk Protein or Casein Supplementation on Muscle Fatigue Following Resistance Training Program: A Randomized, Double-Blind, Placebo-Controlled Study. J. Int. Soc. Sports Nutr..

[101] Snijders T., Res P.T., Smeets J.S.J., van Vliet S., van Kranenburg J., Maase K., Verdijk L.B., van Loon L.J.C. (2015). Protein Ingestion before Sleep Increases Muscle Mass and Strength Gains during Prolonged Resistance-Type Exercise Training in Healthy Young Men. J. Nutr..

[102] Souza-Junior T.P., Willardson J.M., Bloomer R., Leite R.D., Fleck S.J., Oliveira P.R., Simão R. (2011). Strength and Hypertrophy Responses to Constant and Decreasing Rest Intervals in Trained Men Using Creatine Supplementation. J. Int. Soc. Sports Nutr..

[103] Cribb P.J., Williams A.D., Hayes A. (2007). Effects of Whey Isolate, Creatine, and Resistance Training on Muscle Hypertrophy. Med. Sci. Sports Exerc..

[104] Tritto A.C., Bueno S., Rodrigues R.M.P., Gualano B., Roschel H., Artioli G.G. (2019). Negligible Effects of β-Hydroxy-β-Methylbutyrate Free Acid and Calcium Salt on Strength and Hypertrophic Responses to Resistance Training: A Randomized, Placebo-Controlled Study. Int. J. Sport Nutr. Exerc. Metab..

[105] Morton R.W., Murphy K.T., McKellar S.R., Schoenfeld B.J., Henselmans M., Helms E., Aragon A.A., Devries M.C., Banfield L., Krieger J.W. (2018). A Systematic Review, Meta-Analysis and Meta-Regression of the Effect of Protein Supplementation on Resistance Training-Induced Gains in Muscle Mass and Strength in Healthy Adults. Br. J. Sports Med..

[106] Antonio J., Ellerbroek A., Silver T., Vargas L., Peacock C. (2022). The Effects of Consuming a High Protein Diet (4.4 g/kg/d) on Body Composition in Resistance-Trained Individuals. J. Int. Soc. Sports Nutr..

[107] Antonio J., Ellerbroek A., Silver T., Orris S., Scheiner M., Gonzalez A., Peacock C. (2022). A High Protein Diet (3.4 g/kg/d) Combined with a Heavy Resistance Training Program Improves Body Composition in Healthy Trained Men and Women—A Follow-Up Investigation. J. Int. Soc. Sports Nutr..

[108] Candow D.G., Vogt E., Johannsmeyer S., Forbes S.C., Farthing J.P. (2015). Strategic Creatine Supplementation and Resistance Training in Healthy Older Adults. Appl. Physiol. Nutr. Metab..

[109] Antonio J., Ellerbroek A., Silver T., Vargas L., Tamayo A., Buehn R., Peacock C.A. (2016). A High-Protein Diet Has No Harmful Effects: A One-Year Crossover Study in Resistance-Trained Men. J. Nutr. Metab..

[110] Haun C.T., Vann C.G., Mobley C.B., Roberson P.A., Osburn S.C., Holmes H.M., Mumford P.M., Romero M.A., Young K.C., Moon J.R. (2018). Effects of Graded Whey Supplementation During Extreme-Volume Resistance Training. Front. Nutr..

[111] Trommelen J., Kouw I.W.K., Holwerda A.M., Snijders T., Halson S.L., Rollo I. (2018). Presleep Dietary Protein-Derived Amino Acids Are Utilized for De Novo Myofibrillar Protein Synthesis During Overnight Recovery from Exercise in Healthy Older Men. Am. J. Physiol. Endocrinol. Metab..

[112] Haun C.T., Vann C.G., Osburn S.C., Mumford P.W., Roberson P.A., Romero M.A., Fox C.D., Johnson C.A., Parry H.A., Kavazis A.N. (2019). Muscle Fiber Hypertrophy in Response to 6 Weeks of High-Volume Resistance Training in Trained Young Men Is Largely Attributed to Sarcoplasmic Hypertrophy. PLoS ONE.

[113] Willoughby D.S., Stout J.R., Wilborn C.D. (2007). Effects of Resistance Training and Protein Plus Amino Acid Supplementation on Muscle Anabolism, Mass, and Strength. Amino Acids.

