# Supplementary material for: Nutritional Supplements for Muscle Hypertrophy: Mechanisms and Morphology—Focused Evidence
Source: Nutrients. 2025 Nov 18;17(22):3603. doi: 10.3390/nu17223603 (PMC12655760; doi:10.3390/nu17223603)
Supplement: Supplementary file 1 [file nutrients-17-03603-s001.zip › nutrients-3925459-supplementary.pdf]

Supplementary Materials

Table S1. Randomized controlled trials investigating nutritional supplementation and muscle hypertrophy assessed with imaging methods in adults

ID	Study (Author, Year)	Population (N, training status)	Supplement (dose / timing)	Comparator	Training (duration, freq)	Imaging method (site)	Main hypertrophy finding
**1**	Reidy et al., 2016	Healthy young adults (mixed sexes, untrained), N=40	Whey protein 22 g/day, peri-exercise	Maltodextrin	12 wk, 3×/wk	Ultrasound – vastus lateralis	Training effect only; no extra benefit from whey
**2**	Mobley et al., 2017	College-aged men, N=48, untrained	Whey, soy, leucine blend 2×/day	Isoenergetic placebo	12 wk, 3×/wk	Ultrasound – vastus lateralis	VL thickness ↑ similarly in all groups
**3**	Joy et al., 2013	Recreationally trained men, N=16	Whey vs rice protein 48 g post-exercise	Rice protein	8 wk, split RT	Ultrasound – biceps, VL CSA	Similar hypertrophy with whey and rice
**4**	Lynch et al., 2020	Men & women, untrained, N=48	Soy vs whey (leucine-matched)	Whey	12 wk, 3×/wk	Ultrasound – vastus lateralis	No differences; training effect only
**5**	Babault et al., 2015	Young untrained men, N=60	Pea protein 25 g ×2/day	Whey or placebo	12 wk, upper-body RT	Ultrasound – biceps brachii	Pea ≈ whey; pea > placebo in weaker subgroup
**6**	Bridge et al., 2019	Untrained men, N=30	Greek yogurt (~20 g protein/serving)	Isoenergetic pudding	12 wk, 3×/wk + plyometrics	Ultrasound – biceps	Yogurt > pudding for MT and lean mass
**7**	Sharp et al., 2018	Recreational adults, N=30	Hydrolyzed beef protein + CHO	CHO only	8 wk RT	Ultrasound – limb MT	Similar MT between groups
**8**	Naclerio et al., 2017	Recreational adults, N=48	Beef vs whey vs CHO (~20 g)	Carbohydrate	10 wk RT	Ultrasound – arm/leg MT	No between-group differences
**9**	Farup et al., 2014	Young adults, N=30	Whey hydrolysate high-leucine	Carbohydrate	12 wk unilateral RT	MRI – quadriceps CSA	Greater CSA gains with whey hydrolysate
**10**	Vieillevoye et al., 2010	Young men, N=20	EAA + CHO 15 g post-ex	CHO	12 wk RT	Ultrasound – gastrocnemius	EAA+CHO > CHO for muscle architecture
**11**	Banaszek et al., 2019	Recreational adults (HIFT), N=29	Whey vs pea protein daily	Placebo	8 wk HIFT	Ultrasound – quad & arm MT	MT ↑ all groups; no supplement effect
**12**	Jacinto et al., 2022	Young adults, N=22	Whey 35 g vs collagen 35 g	Collagen	10 wk, 3×/wk	Ultrasound – biceps, VL	Whey > collagen for MT
**13**	Hartman et al., 2007	Novice young men, N=56	Milk vs soy vs CHO post-ex	CHO	12 wk RT	MRI – quadriceps volume	Milk > soy & CHO for hypertrophy
**14**	Chilibeck et al., 2004	Young adults, N=20	Creatine post-exercise	Placebo	12 wk unilateral RT	Ultrasound – elbow flexors, quads	Creatine > placebo for MT
**15**	Candow et al., 2011	Active adults, N=38	Creatine (0.1–0.15 g/kg)	Placebo	6 wk, 3×/wk	Ultrasound – elbow	Creatine > placebo for MT

						flexors, quads	
**16**	Mills et al., 2020	Trained adults, N=22	Creatine intra-workout	Placebo	6 wk, 5×/wk	Ultrasound – arms, legs	No group differences; RT effect only
**17**	Pakulak et al., 2022	Resistance-trained, N=13	Creatine pre-workout	Placebo	6 wk, 4×/wk	Ultrasound – quads	Creatine > placebo for MT
**18**	Schoenfeld et al., 2017	Trained men, N=21	25 g protein pre vs post	Alternate timing	10 wk RT	Ultrasound – forearm flexors, VL	Similar gains; timing not critical
**19**	Joy et al., 2014	Trained adults, N=28	Phosphatidic acid 750 mg/day	Placebo	8 wk RT	Ultrasound – quads	Modest ↑ with PA
**20**	Gonzalez et al., 2017	Trained men, N=28	PA 750 mg/day	Placebo	8 wk RT	Ultrasound – quads, arms	No differences vs placebo
**21**	Andre et al., 2016	Trained men, N=28	PA 250–375 mg/day	Placebo	8 wk RT	Ultrasound – regional MT	No clear effect of PA
**22**	Escalante et al., 2016	Recreational adults, N=28	Multi-ingredient (PA + leucine)	Placebo	8 wk RT	Ultrasound – quads	LBM ↑ in supplement vs placebo
**23**	Wilson et al., 2014	Trained, N=40	HMB-FA 3 g/day	Placebo	12 wk RT	Ultrasound – quads	Greater quad MT with HMB-FA
**24**	Lowery et al., 2016	Trained, N=40	HMB-FA + ATP	Placebo	12 wk RT	Ultrasound – quads	HMB-FA > placebo for MT
**25**	Townsend et al., 2022	Athletes, N=25	Red spinach extract (nitrate)	Placebo	11 wk RT	Ultrasound – RF, VL	MT ↑ both; no supplement effect
**26**	Jerger et al., 2022	Young men, N=30	Collagen peptides 5 g/day	Placebo	14 wk RT	MRI + US – RF CSA	Tendon improved; small MT changes
**27**	Balshaw et al., 2023	Adults, N=25	Collagen peptides	Placebo	12 wk RT	MRI + US	Tendon effects; little hypertrophy
**28**	Lee et al., 2023	Elite female soccer, N=28	Collagen + vit C	Carb placebo	10 wk RT	Ultrasound – VL, tendon	Tendon improved; MT similar
**29**	Michel et al., 2022	Adults, N=36	Protein intake targets	Different protein doses	12 wk RT	Ultrasound – VL	Higher protein ↑ MT modestly
**30**	Fraschetti et al., 2025	Young men, N=30	Greek yogurt	Carb control	12 wk RT+plyo	Ultrasound – multi-site	Yogurt > control for MT
**31**	Joy et al., 2018	Trained adults, N=13	Casein daytime vs pre-sleep	Alternate timing	10 wk RT	Ultrasound – quads	No timing effect
**32**	Babault et al., 2014	Active men, N=44	Soluble milk protein vs casein	Placebo	10 wk RT	Ultrasound – MT	Protein groups > placebo
**33**	Snijders et al., 2015	Young men, N=44	Pre-sleep protein 27.5 g	Placebo	12 wk RT	CT – quads CSA	Protein > placebo for CSA
**34**	Souza-Junior et al., 2011	Trained men, N=22	Creatine + RT	Placebo	8 wk RT	Ultrasound – arms, legs	Creatine > placebo for CSA
**35**	Cribb et al., 2007	Resistance-trained, N=23	Whey protein + creatine + glucose	Glucose	10 wk RT	MRI – quad CSA	Combo > glucose for hypertrophy
**36**	Trtto et al., 2019	Young men, N=44	HMB-FA 3 g/day	Placebo	12 wk RT	VL/RF muscles thickness	No effect of HMB-FA
**37**	Morton et al., 2018	Trained men, N=49	Whey vs soy (matched protein)	Soy	12 wk RT	MRI – quad CSA	Both ↑; whey ≈ soy

**38**	Antonio et al., 2022	Trained men, N=30	Very high protein (>3 g/kg/d)	Normal protein	8 wk RT	DXA + US – quads	No adverse effect; MT similar
**39**	Antonio et al., 2022	Trained, N=48	High protein diet	Normal protein	12 wk RT	Ultrasound – quads	No diff in MT
**40**	Candow et al., 2015	Middle-aged men, N=30	Creatine 0.1 g/kg/d	Placebo	10 wk RT	Ultrasound – arms, legs	Creatine > placebo for MT
**41**	Antonio et al., 2016	Trained men, N=46	High protein intake (3.3 g/kg)	Habitual intake	8 wk RT	Ultrasound – biceps, quads	No adverse effect; MT similar
**42**	Haun et al., 2018	Resistance-trained, N=30	Multi-ingredient (PRO+CHO+CREA)	Placebo	6 wk RT	Ultrasound – quads	Supplement > placebo early phase
**43**	Churchward-Venne et al., 2014	Young men, N=48	Whey + leucine vs whey alone	Whey	12 wk RT	MRI – quads CSA	No added effect of leucine
**44**	Trommelen et al., 2018	Young men, N=40	Casein at night	Placebo	12 wk RT	MRI – quads CSA	Pre-sleep protein > placebo
**45**	Haun et al., 2019	Trained men, N=40	Multi-ingredient with PA	Placebo	8 wk RT	Ultrasound – quads	Small effect, not robust
**46**	Willoughby et al., 2007	Resistance-trained men, N=23	Creatine + whey protein	Placebo	10 wk RT	MRI – quads CSA	Creatine+protein > placebo

Notes: All trials included were randomized controlled trials in adults undergoing resistance training with nutritional supplementation, with muscle hypertrophy assessed by imaging methods (ultrasound, MRI, or CT). Only studies in adult populations were retained; trials in elderly, adolescents, or clinical patients were excluded. Abbreviations: VL, vastus lateralis; RF, rectus femoris; CSA, cross-sectional area; MT, muscle thickness; RT, resistance training; CHO, carbohydrate.

References

1. Reidy, P.T.; Borack, M.S.; Markofski, M.M.; Dickinson, J.M.; Deer, R.R.; Husaini, S.H.; Walker, D.K.; Igbinigie, S.; Robertson, S.M.; Cope, M.B.; et al. Protein Supplementation Has Minimal Effects on Muscle Adaptations during Resistance Exercise Training in Young Men: A Double-Blind Randomized Clinical Trial. J. Nutr. **2016**, 146, 1660–1669. https://doi.org/10.3945/jn.116.231803
2. Mobley, C.B.; Haun, C.T.; Roberson, P.A.; Mumford, P.W.; Kephart, W.C.; Romero, M.A.; Osburn, S.C.; Vann, C.G.; Young, K.C.; Beck, D.T.; et al. Effects of Whey, Soy or Leucine Supplementation with 12 Weeks of Resistance Training on Strength, Body Composition, and Skeletal Muscle and Adipose Tissue Histological Attributes in College-Aged Males. Nutrients **2017**, 9, 972. https://doi.org/10.3390/nu9090972
3. Joy, J.M.; Lowery, R.P.; Wilson, J.M.; Purpura, M.; De Souza, E.O.; Wilson, S.M.; Kalman, D.S.; Dudeck, J.E.; Jäger, R. The Effects of 8 Weeks of Whey or Rice Protein Supplementation on Body Composition and Exercise Performance. Nutr. J. **2013**, 12, 86. https://doi.org/10.1186/1475-2891-12-86
4. Lynch, H.M.; Wharton, C.; Johnston, C.S.; Kris-Etherton, P.M.; Post, R.E.; Parkinson, A.L.; Little, R.B.; Most, M.; West, S.G.; Armamento-Villareal, R.; et al. No Significant Differences in Muscle Growth and Strength Development When Consuming Soy and Whey Protein Supplements Matched for Leucine Following a 12-Week Resistance Training Program in Men and Women: A Randomized Trial. Int. J. Environ. Res. Public Health **2020**, 17, 3871. https://doi.org/10.3390/ijerph17113871
5. Babault, N.; Païzis, C.; Deley, G.; Guérin-Deremaux, L.; Saniez, M.H.; Lefranc-Millot, C.; Allaert, F.A. Pea Proteins Oral Supplementation Promotes Muscle Thickness Gains during Resistance Training: A Double-Blind, Randomized, Placebo-Controlled Clinical Trial vs. Whey Protein. J. Int. Soc. Sports Nutr. **2015**, 12, 3. https://doi.org/10.1186/s12970-014-0064-5
6. Bridge, A.; Brown, J.; Snider, H.; Nasato, M.; Prapavessis, H. Greek Yogurt and 12 Weeks of Exercise Training on Strength, Muscle Thickness, and Body Composition in Lean, Untrained, University-Aged Males. *Front. Nutr.* **2019**, *6*, 55. https://doi.org/10.3389/fnut.2019.00055
7. Sharp, M.H.; Lowery, R.P.; Shields, K.A.; Lane, J.R.; Gray, J.L.; Partl, J.M.; Hayes, D.W.; Wilson, G.J.; Hollmer, C.A.; Minivich, J.R.; Wilson, J.M**.** The Effects of Beef, Chicken, or Whey Protein after Workout

on Body Composition and Muscle Performance. *J. Strength Cond. Res.* **2018**, *32*(8), 2233–2242. https://doi.org/10.1519/JSC.0000000000001936

8. Naclerio, F.; Seijo, M.; Larumbe-Zabala, E.; Earnest, C.P**.** Carbohydrates Alone or Mixing With Beef or Whey Protein Promote Similar Training Outcomes in Resistance-Training Males: A Double-Blind, Randomized Controlled Clinical Trial. *Int. J. Sport Nutr. Exerc. Metab.* **2017**, *27*(5), 408–420. https://doi.org/10.1123/ijsnem.2017-0003
9. Farup, J.; Rahbek, S.K.; Vendelbo, M.H.; Matzon, A.; Hindhede, J.; Bejder, A.; Ringgaard, S.; Vissing, K. High-Leucine Whey Protein Hydrolysate Augments Muscle and Tendon Hypertrophy Following 12 Weeks of Resistance Training—Irrespective of Contraction Mode. *Scand. J. Med. Sci. Sports* **2014**, *24*, 788–798. https://doi.org/10.1111/sms.12083
10. Vieillevoye, S.; Poortmans, J.R.; Duchateau, J.; Carpentier, A. Effects of a Combined Essential Amino Acids/Carbohydrate Supplementation on Muscle Mass, Architecture and Maximal Strength Following Heavy-Load Training. *Eur. J. Appl. Physiol.* **2010**, *110*, 479–488. https://doi.org/10.1007/s00421-010-1520-9
11. Banaszek, A.; Townsend, J.R.; Bender, D.; Vantrease, W.C.; Marshall, A.C.; Johnson, K.D. The Effects of Whey vs. Pea Protein on Physical Adaptations Following 8-Weeks of High-Intensity Functional Training (HIFT): A Pilot Study. Sports **2019**, 7, 12. https://doi.org/10.3390/sports7010012
12. Jacinto JL, Nunes JP, Gorissen SHM, Capel DMG, Bernardes AG, Ribeiro AS, Cyrino ES, Phillips SM, Aguiar AF. Whey Protein Supplementation Is Superior to Leucine-Matched Collagen Peptides to Increase Muscle Thickness During a 10-Week Resistance Training Program in Untrained Young Adults. Int J Sport Nutr Exerc Metab. **2022** May 1;32(3):133-143. DOI: 10.1123/ijsnem.2021-0265
13. Hartman, J.W.; Tang, J.E.; Wilkinson, S.B.; Tarnopolsky, M.A.; Lawrence, R.L.; Fullerton, A.V.; Phillips, S.M. Consumption of Fat-Free Fluid Milk after Resistance Exercise Promotes Greater Lean Mass Accretion than Does Consumption of Soy or Carbohydrate in Young, Novice, Male Weightlifters. Am. J. Clin. Nutr. **2007**, 86, 373–381. https://doi.org/10.1093/ajcn/86.2.373
14. Chilibeck, P.D.; Stride, D.; Farthing, J.P.; Burke, D.G. Effect of Creatine Ingestion after Exercise on Muscle Thickness in Males and Females. *Med. Sci. Sports Exerc.* **2004**, *36*, 1781–1788. PMID:15595301.
15. Candow, D.G.; Chilibeck, P.D.; Burke, D.G.; Mueller, K.D.; Lewis, J.D. Effect of Different Frequencies of Creatine Supplementation on Muscle Size and Strength in Young Adults. *J. Strength Cond. Res.* **2011**, *25*(7), 1831–1838. https://doi.org/10.1519/JSC.0b013e3181e7419a
16. Mills, S.; Candow, D.G.; Forbes, S.C.; Neary, J.P.; Ormsbee, M.J.; Antonio, J. Effects of Creatine Supplementation during Resistance Training Sessions in Physically Active Young Adults. *Nutrients* **2020**, *12*(6), 1880. https://doi.org/10.3390/nu12061880
17. Pakulak, A.; Muddle, T.W.D.; Rollo, I.; Galloway, S.D.R.; Pritchard, H.J.; Tallis, J. Effects of Creatine and Caffeine Supplementation during Resistance Training on Body Composition, Strength, Endurance, RPE and Fatigue in Trained Young Adults. *J. Diet. Suppl.* **2022**, *19*(5), 514–529. https://doi.org/10.1080/19390211.2021.1904085
18. Schoenfeld, B.J.; Aragon, A.A.; Wilborn, C.; Urbina, S.; Hayward, S.; Krieger, J. Pre- versus Post-Exercise Protein Intake Has Similar Effects on Muscular Adaptations. *PeerJ* **2017**, *5*, e2825. https://doi.org/10.7717/peerj.2825
19. Joy, J.M.; Lowery, R.P.; Wilson, J.M.; Purpura, M.; De Souza, E.O.; McDonnell, E.; Wilson, S.M.C.; Kalman, D.S.; Dudeck, J.E.; Jäger, R. Phosphatidic Acid Enhances mTOR Signaling and Resistance Exercise Training Adaptations in Human Skeletal Muscle. *Nutr. Metab.* **2014,** *11*, 29. https://doi.org/10.1186/1743-7075-11-29
20. Gonzalez, A.M.; Sell, K.M.; Ghigiarelli, J.J.; Kelly, C.F.; Shone, E.W.; Accetta, M.R.; Baum, J.B.; Mangine, G.T. Effects of Phosphatidic Acid Supplementation on Muscle Thickness and Strength in Resistance-Trained Men. *Appl. Physiol. Nutr. Metab.* **2017**, *42*(4), 443–448. https://doi.org/10.1139/apnm-2016-0564
21. Andre, T.L.; Gann, J.J.; McKinley-Barnard, S.K.; Song, J.J.; Willoughby, D.S. Eight Weeks of Phosphatidic Acid Supplementation in Conjunction with Resistance Training Does Not Differentially Affect Body Composition and Muscle Strength in Resistance-Trained Men. *J. Sports Sci. Med.* **2016**, *15*(3), 532–539. PMID: 27803633
22. Escalante, G.; Hoffman, J.; Almeda, J.; et al. The Effects of Phosphatidic Acid Supplementation on Strength, Body Composition, Muscular Endurance, Power, Agility, and Vertical Jump in Resistance Trained Men. *J. Int. Soc. Sports Nutr.* **2016**, *13*, 24. https://doi.org/10.1186/s12970-016-0135-x

23. Wilson, J.M.; Lowery, R.P.; Joy, J.M.; Andersen, J.C.; Wilson, S.M.C.; Stout, J.R.; Duncan, N.; Fuller, J.C.; Baier, S.M.; Naimo, M.A.; Rathmacher, J.A. The Effects of 12 Weeks of Beta-Hydroxy-Beta-Methylbutyrate Free Acid Supplementation on Muscle Mass, Strength, and Power in Resistance-Trained Individuals: A Randomized, Double-Blind, Placebo-Controlled Study. *Eur. J. Appl. Physiol.* **2014**, *114*(6), 1217–1227. https://doi.org/10.1007/s00421-014-2854-5
24. Lowery, R.P.; Joy, J.M.; Rathmacher, J.A.; Baier, S.M.; Fuller, J.C., Jr.; Shelley, M.C., II; Jäger, R.; Purpura, M.; Wilson, S.M.C.; Wilson, J.M. Interaction of Beta-Hydroxy-Beta-Methylbutyrate Free Acid and Adenosine Triphosphate on Muscle Mass, Strength, and Power in Resistance-Trained Individuals. *J. Strength Cond. Res.* **2016**, *30*(7), 1843–1854. (Publisher page) https://journals.lww.com/nsca-jscr/fulltext/2016/07000/interaction_of_beta_hydroxy_beta_methylbutyrate.7.aspx
25. Townsend, J.R.; Hart, T.L.; Haynes, J.T.; et al. Influence of Dietary Nitrate Supplementation on Physical Performance and Body Composition Following Offseason Training in Division I Athletes. *J. Diet. Suppl.* **2022**, *19*(4), 534–549. https://doi.org/10.1080/19390211.2021.1900482
26. Jerger, S.; Bohm, S.; Marzilger, R.; Mersmann, F.; Arampatzis, A. Effects of Specific Collagen Peptide Supplementation on Tendon and Muscle Adaptations to High-Load Resistance Training: A Randomized Controlled Trial. *Scand. J. Med. Sci. Sports* **2022**, *32*(8), 1212–1226. https://doi.org/10.1111/sms.14164
27. Balshaw TG, Funnell MP, McDermott E, Maden-Wilkinson TM, Abela S, Quteishat B, Edsey M, James LJ, Folland JP. The effect of specific bioactive collagen peptides on function and muscle remodeling during human resistance training. Acta Physiol (Oxf). **2023** Feb;237(2):e13903. DOI: 10.1111/apha.13903
28. Lee, J.; Bridge, J.E.; Clark, D.R.; Stewart, C.E.; Erskine, R.M. Collagen Supplementation Augments Changes in Patellar Tendon Properties in Female Soccer Players. *Front. Physiol.* **2023**, *14*, 1089971. https://doi.org/10.3389/fphys.2023.1089971
29. Michel, J.M.; Lievense, K.K.; Norton, S.C.; Costa, J.V.; Alphin, K.H.; Bailey, L.A.; Miller, G.D. The Effects of Graded Protein Intake in Conjunction with Resistance Training on Muscle Mass, Strength, and Physical Function in Older Adults: A Randomized Trial. *Nutrients* **2022**, *14*(13), 2739. https://doi.org/10.3390/nu14132739
30. Fraschetti, E.C.; Abdul-Sater, A.A.; Perry, C.G.R.; Josse, A.R. *Resistance Exercise Training and Greek Yogurt Consumption Modulate Markers of Systemic Inflammation in Healthy Young Males—A Secondary Analysis of a Randomized Controlled Trial.* Nutrients **2025**, 17(17), 2816. https://doi.org/10.3390/nu17172816
31. Joy, J.M.; Vogel, R.M.; DiMarco, N.; et al. Daytime and Nighttime Casein Supplements Similarly Increase Muscle Size and Strength in Response to Resistance Training. *J. Int. Soc. Sports Nutr.* **2018**, *15*, 24. https://doi.org/10.1186/s12970-018-0228-9
32. Babault, N.; Deley, G.; Le Ruyet, P.; Morgan, F.; Allaert, F.A. Effects of Soluble Milk Protein or Casein Supplementation on Muscle Fatigue Following Resistance Training Program: A Randomized, Double-Blind, Placebo-Controlled Study. J. Int. Soc. Sports Nutr. **2014**, 11, 36. https://doi.org/10.1186/1550-2783-11-36
33. Snijders, T.; Res, P.T.; Smeets, J.S.J.; van Vliet, S.; van Kranenburg, J.; Maase, K.; Verdijk, L.B.; van Loon, L.J.C. Protein Ingestion before Sleep Increases Muscle Mass and Strength Gains during Prolonged Resistance-Type Exercise Training in Healthy Young Men. *J. Nutr.* **2015**, *145*(6), 1178–1184. https://doi.org/10.3945/jn.114.208371
34. Souza-Junior, T.P.; Willardson, J.M.; Bloomer, R.; Leite, R.D.; Fleck, S.J.; Oliveira, P.R.; Simão, R. *Strength and Hypertrophy Responses to Constant and Decreasing Rest Intervals in Trained Men Using Creatine Supplementation.* J. Int. Soc. Sports Nutr. **2011**, 8, 17. https://doi.org/10.1186/1550-2783-8-17
35. Cribb, P.J.; Williams, A.D.; Hayes, A. Effects of Whey Isolate, Creatine, and Resistance Training on Muscle Hypertrophy. *Med. Sci. Sports Exerc.* **2007**, *39*(11), 1960–1968. https://pubmed.ncbi.nlm.nih.gov/17277594
36. Tritto, A.C.; Bueno, S.; Rodrigues, R.M.P.; Gualano, B.; Roschel, H.; Artioli, G.G. Negligible Effects of β-Hydroxy-β-Methylbutyrate Free Acid and Calcium Salt on Strength and Hypertrophic Responses to Resistance Training: A Randomized, Placebo-Controlled Study. *Int. J. Sport Nutr. Exerc. Metab.* **2019**, *29*(5), 505–511. https://doi.org/10.1123/ijsnem.2018-0337
37. Morton, R.W.; Murphy, K.T.; McKellar, S.R.; Schoenfeld, B.J.; Henselmans, M.; Helms, E.; Aragon, A.A.; Devries, M.C.; Banfield, L.; Krieger, J.W.; et al. A Systematic Review, Meta-Analysis and Meta-Regression of the Effect of Protein Supplementation on Resistance Training-Induced Gains in Muscle

Mass and Strength in Healthy Adults. Br. J. Sports Med. **2018**, 52, 376–384. https://doi.org/10.1136/bjsports-2017-097608

38. Antonio, J.; Ellerbroek, A.; Silver, T.; Vargas, L.; Peacock, C. The Effects of Consuming a High Protein Diet (4.4 g/kg/d) on Body Composition in Resistance-Trained Individuals. *J. Int. Soc. Sports Nutr.* **2022**, *11*, 19. https://doi.org/10.1186/1550-2783-11-19
39. Antonio, J.; Ellerbroek, A.; Silver, T.; Orris, S.; Scheiner, M.; Gonzalez, A.; Peacock, C. A High Protein Diet (3.4 g/kg/d) Combined with a Heavy Resistance Training Program Improves Body Composition in Healthy Trained Men and Women—A Follow-Up Investigation. *J. Int. Soc. Sports Nutr.* **2022**, *12*, 39. https://doi.org/10.1186/s12970-015-0100-0
40. Candow, D.G.; Vogt, E.; Johannsmeyer, S.; Forbes, S.C.; Farthing, J.P. Strategic Creatine Supplementation and Resistance Training in Healthy Older Adults. *Appl. Physiol. Nutr. Metab.* **2015**, *40*(7), 689–694. https://doi.org/10.1139/apnm-2014-0498
41. Antonio, J.; Ellerbroek, A.; Silver, T.; Vargas, L.; Tamayo, A.; Buehn, R.; Peacock, C.A. A High-Protein Diet Has No Harmful Effects: A One-Year Crossover Study in Resistance-Trained Men. *J. Nutr. Metab.* **2016** https://doi.org/10.1155/2016/9104792.
42. Haun CT, Vann CG, Mobley CB, Roberson PA, Osburn SC, Holmes HM, Mumford PM, Romero MA, Young KC, Moon JR, Gladden LB, Arnold RD, Israetel MA, Kirby AN, Roberts MD. Effects of Graded Whey Supplementation During Extreme-Volume Resistance Training. *Front Nutr.* **2018** Sep 11;5:84. DOI: 10.3389/fnut.2018.00084
43. Churchward-Venne, T.A.; Breen, L.; Di Donato, D.M.; et al. *Leucine Supplementation of a Low-Protein Mixed Macronutrient Beverage Enhances Myofibrillar Protein Synthesis in Young Men: A Double-Blind, Randomized Trial.* Am. J. Clin. Nutr. **2014**, 99(2), 276–286. https://doi.org/10.3945/ajcn.113.068775
44. Trommelen, J.; Kouw, I.W.K.; Holwerda, A.M.; et al. *Presleep Dietary Protein-Derived Amino Acids Are Utilized for De Novo Myofibrillar Protein Synthesis During Overnight Recovery from Exercise in Healthy Older Men.* Am. J. Physiol. Endocrinol. Metab. **2018**, 314(5), E457–E467. https://doi.org/10.1152/ajpendo.00273.2016
45. Haun, C.T.; Vann, C.G.; Osburn, S.C.; Mumford, P.W.; Roberson, P.A.; Romero, M.A.; et al. Muscle Fiber Hypertrophy in Response to 6 Weeks of High-Volume Resistance Training in Trained Young Men Is Largely Attributed to Sarcoplasmic Hypertrophy. *PLoS ONE* **2019**, *14*(6), e0215267. https://doi.org/10.1371/journal.pone.0215267
46. Willoughby, D.S.; Stout, J.R.; Wilborn, C.D. Effects of Resistance Training and Protein Plus Amino Acid Supplementation on Muscle Anabolism, Mass, and Strength. *Amino Acids* **2007**, *32*(4), 467–477. https://doi.org/10.1007/s00726-006-0398-7
